# A Neurodynamic Model of Feature-Based Spatial Selection

**DOI:** 10.3389/fpsyg.2018.00417

**Published:** 2018-03-28

**Authors:** Mateja Marić, Dražen Domijan

**Affiliations:** Department of Psychology, Faculty of Humanities and Social Sciences, University of Rijeka, Rijeka, Croatia

**Keywords:** boolean map, feature-based attention, lateral inhibition, neural network, winner-take-all

## Abstract

Huang and Pashler (2007) suggested that feature-based attention creates a special form of spatial representation, which is termed a Boolean map. It partitions the visual scene into two distinct and complementary regions: selected and not selected. Here, we developed a model of a recurrent competitive network that is capable of state-dependent computation. It selects multiple winning locations based on a joint top-down cue. We augmented a model of the WTA circuit that is based on linear-threshold units with two computational elements: dendritic non-linearity that acts on the excitatory units and activity-dependent modulation of synaptic transmission between excitatory and inhibitory units. Computer simulations showed that the proposed model could create a Boolean map in response to a featured cue and elaborate it using the logical operations of intersection and union. In addition, it was shown that in the absence of top-down guidance, the model is sensitive to bottom-up cues such as saliency and abrupt visual onset.

## Introduction

In the literature on visual attention, significant progress has been made in characterizing the principles of selection. Visual attention can be allocated flexibly to a circumscribed region of space, the whole object or feature dimensions such as color and orientation (Nobre and Kastner, 2014). Indeed, early work suggested that a restricted circular region of space is a representational format of attentional selection. Posner (1980) proposed that attention operates like a spotlight that highlights a single circular region of space with a fixed radius. All locations that fall inside the spotlight are selected, and everything outside is left out. An extension of this proposal, which is called the zoom-lens model, suggests that the spotlight of attention can change its radius depending on the spatial resolution that one wants to achieve (Eriksen and St. James, 1986). If high resolution is required, the spotlight can be narrowed to capture details in the selected region, whereas the radius of the spotlight can be widened when a lower resolution is sufficient.

Other studies point to an object as a unit of selection. Duncan (1984) showed that it is easier to report two attributes if they appear on the same object, relative to the scenario in which each attribute appears on a different object. This finding implies that the object is selected as a whole and has been replicated many times using different stimuli and behavioral paradigms (Scholl, 2001). This effect cannot be explained by spatial attention because objects were spatially superimposed, that is, they shared the same locations. More recently, it was shown that attention can also be allocated to a visual feature such as color or direction of motion independent of spatial location (Saenz et al., 2002, 2003). Single-unit recordings have shown that feature-based attention is accompanied by the global location-independent modulation of neural response in a range of areas in the visual cortex. Attentional modulation was described as a multiplicative gain change that increases responses of neurons that are selective to attended feature values and decreases responses of neurons that are tuned to unattended feature values (Treue and Martinez-Trujillo, 1999; Martinez-Trujillo and Treue, 2004).

Object-based attention, however, is not necessarily detached from spatial representation. There is behavioral and neurophysiological evidence that object-based attention involves selection of all spatial locations that are occupied by the same object. Specifically, it was suggested that attention selects a grouped array of locations (O'Grady and Müller, 2000). In other words, attention spreads from one spatial location along the shape of the object and highlights all locations that belong to the object (Richard et al., 2008; Vatterott and Vecera, 2015). Neurophysiological studies showed that object-based selection is indeed achieved by the spreading of the enhanced firing rate along the shape of the object (Roelfsema, 2006; Roelfsema and de Lange, 2016).

In a similar way, feature-based attention might involve the selection of all locations that are occupied by the same feature value, as shown by Huang and Pashler (2007). They proposed that attention is limited because it may access only one feature value (e.g., red) per dimension (e.g., color) at any given moment. However, the accessed feature value is bound to space in parallel, without capacity limits. Feature-based attention is allocated in space via the formation of a binary or Boolean map. When a conscious decision is made to attend to a specific feature value, the Boolean map indicates all spatial locations that are occupied by the chosen feature value because they are labeled by a positive value (e.g., 1), while all other locations are labeled with zero. In each selection process, selected locations need not be contiguous in space, but they must share the same feature value. After a Boolean map is formed, it is possible to operate on its output by applying the set operations of intersection and union. Recent work suggests that a spatial representation, such as a Boolean map, might mediate perceptual grouping by similarity (Huang, 2015; Yu and Franconeri, 2015). Moreover, the idea has been recently applied successfully in the computer vision literature on developing algorithms for saliency detection (Zhang and Sclaroff, 2016; Qi et al., 2017).

**Figure 1:**
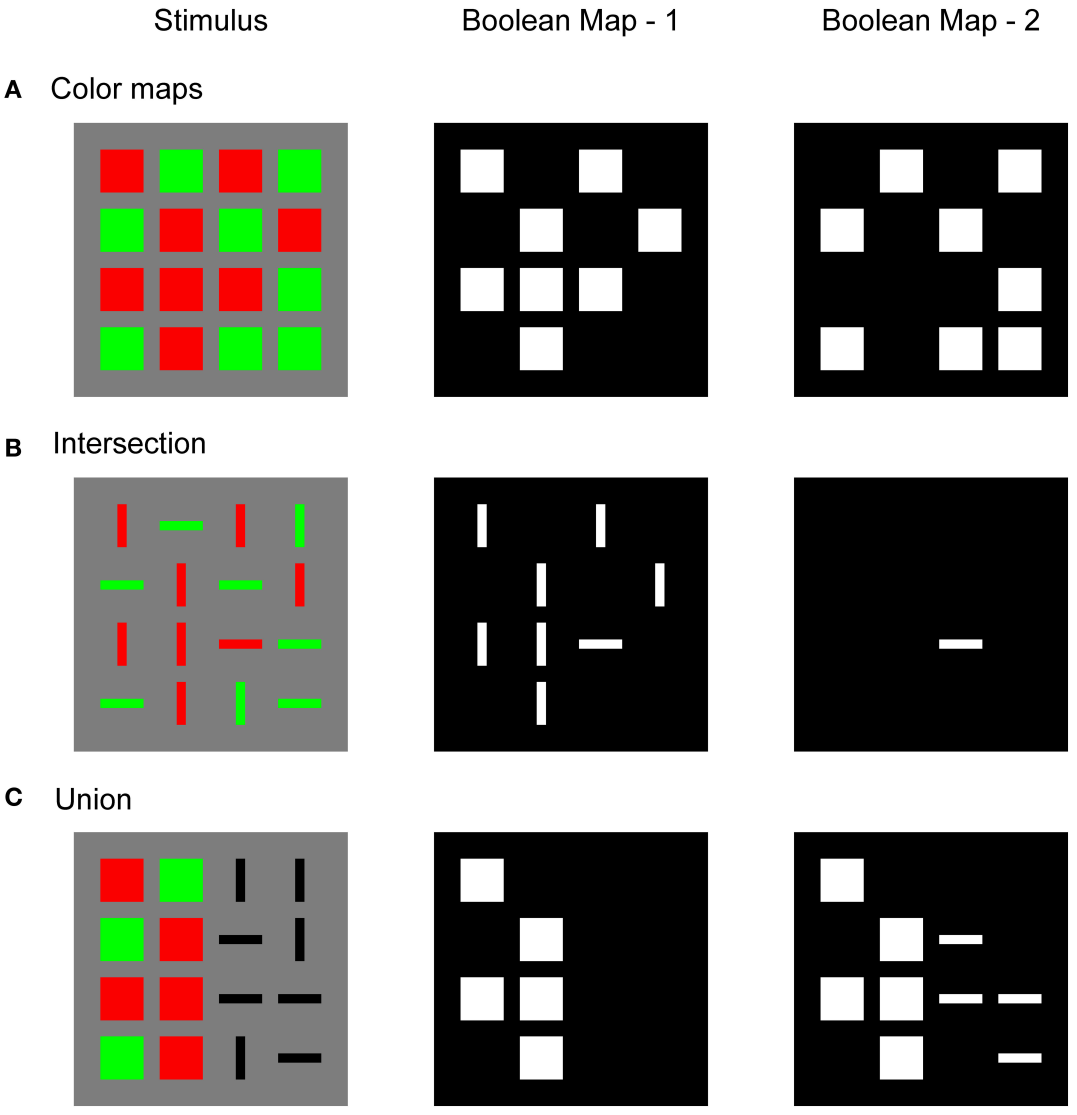
Illustration of the Boolean map that was created in response to the input image (Stimulus) after the first feature cue was applied to the spatial representation (Boolean Map – 1) and after the second feature cue was applied (Boolean Map – 2). **(A)** Boolean maps that were created by two-color cues: red in the first step and green in the second step; **(B)** intersection of two Boolean maps, where red is cued in the first step and horizontal orientation in the second step; **(C)** union of two Boolean maps, where red is cued in the first step and horizontal orientation in the second step.

Figure 1 illustrates a Boolean map that is formed in response to three different stimulus configurations and sequential application of two top-down feature cues. Figure 1A shows a simple stimulus that consists of red and green squares. An observer might attempt to isolate only red or only green items. To do so, a top-down cue should be supplied to the feature map that encodes the desired feature value. For example, when attention is directed to the red color, the top-down cue highlights all locations that are occupied by red squares. The Boolean map picks up on this feature cue and forms a spatial representation in which cued locations are labeled with 1 (white) and non-cued locations are labeled with 0 (black). In terms of a neural network, these labels correspond to the active (excited) and inactive (inhibited) states of the corresponding nodes in the network (Boolean Map – 1). Later, the observer might wish to switch to green color (Boolean Map – 2). Again, in a response to a new feature cue, the Boolean map now shows all locations that are occupied by green squares.

Figure 1B shows a typical stimulus that is used in visual search experiments. It consists of red and green horizontal and vertical bars. The task is to find a red horizontal bar. This is an example of a conjunction search task in which two feature dimensions should be combined to find the target object. According to Huang and Pashler (2007), the conjunction task is solved in two steps. In the first step, a Boolean map is formed by top-down cueing of red items, irrespective of their orientations. In the second step, only horizontal items are cued. However, since red items have already been selected, the second Boolean map will correspond to the intersection of red and horizontal items. There is only one item that satisfies these selection criteria: the target. In this way, visual search is substantially faster compared to the strategy of sequentially visiting each item by moving the attentional spotlight across the visual field. It is also possible to reverse the order of the applied feature cues. In the first step, horizontal items might be cued, and the intersection is formed by highlighting red items in the second step. Importantly, there is behavioral evidence that observers indeed implement such a *subset selection* strategy in conjunction search tasks (Egeth et al., 1984; Kaptein et al., 1995). Moreover, Huang and Pashler (2012) showed that the same strategy is used in the perception of spatial structure in a stimulus that is composed of multiple items that differ in several dimensions.

Figure 1C illustrates an example of the union of two Boolean maps. As in the previous example, the observer starts by cueing red items and creating a Boolean map that consists of a representation of their locations. In the second step, the observer wishes to combine red with horizontal items. Therefore, in the second step, one should cue horizontal items but simultaneously maintain locations of the remaining items in memory. The resulting new Boolean map now represents the locations of all red and all horizontal items that were found in the image. Computing with Boolean maps might not be restricted to only two steps, as Figure 1 suggests. It is possible to incorporate more feature dimensions, such as motion, texture, or size, that can also be engaged in creating Boolean maps that are more complicated.

Feature-based spatial selection, as illustrated by the Boolean map, provides a strong constraint on the computational models of visual attention because it requires simultaneous selection of arbitrarily many locations based on an arbitrary criterion that is set by the observer. Computational models of attention often rely on a winner-take-all (WTA) network to select a single, most salient location from the input image (Itti and Koch, 2000, 2001). The WTA network consists of an array of excitatory nodes that are connected reciprocally with inhibitory interneurons. This anatomical arrangement creates lateral inhibition among excitatory nodes that lead to the selection of a single node that receives maximal input and the suppression of all other nodes, which receive non-maximal input. However, when faced with the input where multiple (potentially many) nodes share the same maximal input level, the typical WTA network tends to suppress all winning nodes due to a strong mutual inhibition among them instead of selecting them together. For example, Usher and Cohen (1999) showed that, under the conditions of strong recurrent excitation and weak lateral inhibition, the WTA network reaches a steady state with multiple active winners. Importantly, activation of the winning nodes decreases linearly toward zero as their quantity increases. In other words, this network design suffers from the capacity limitation. This is a useful property in modeling short-term memory and frontal lobe function (Haarmann and Usher, 2001) but it is inadequate for understanding how the Boolean map might arise in a large retinotopic map, as exemplified by Figure 1.

Another problem is that the dynamics of the WTA network are not sensitive to transient changes in the input amplitude. Due to strong self-excitation and the resulting persistent activity, the WTA network settles into one of its memory states (fixed points). Importantly, each memory state is independent of later inputs. If self-excitation is weakened, the network will become sensitive to input. However, at the same time, it will lose its ability to form a memory state and will behave like a feedforward network (Rutishauser and Douglas, 2009). One way to solve this problem is to apply an external reset signal to the network before a new input is processed (Grossberg, 1980; Kaski and Kohonen, 1994; Itti and Koch, 2000, 2001). However, this is not sufficient in the context of feature-based attention. An intersection or union operation between two Boolean maps requires that the currently active memory state (formed after the first feature cue) be updated by taking into account new input (the second feature cue). Therefore, the dynamics of the WTA network should allow uninterrupted transition between memory states that are governed by external inputs. In other words, the WTA network should be capable of state-dependent computation (Rutishauser and Douglas, 2009).

To summarize, a WTA network that is capable of computing with Boolean maps should simultaneously satisfy two computational constraints:

1. It should be able to select together all locations that share a common feature value. This should be achieved without degrading the representation of the winners.
2. It should exhibit state-dependent computation, in which new inputs are combined with the current memory state to produce a new resultant state (e.g., intersection or union).

Here, we have developed a new WTA network that satisfies these constraints and provides the neural implementation of the Boolean map theory of attention (Huang and Pashler, 2007).

## Model description

**Figure 2:**
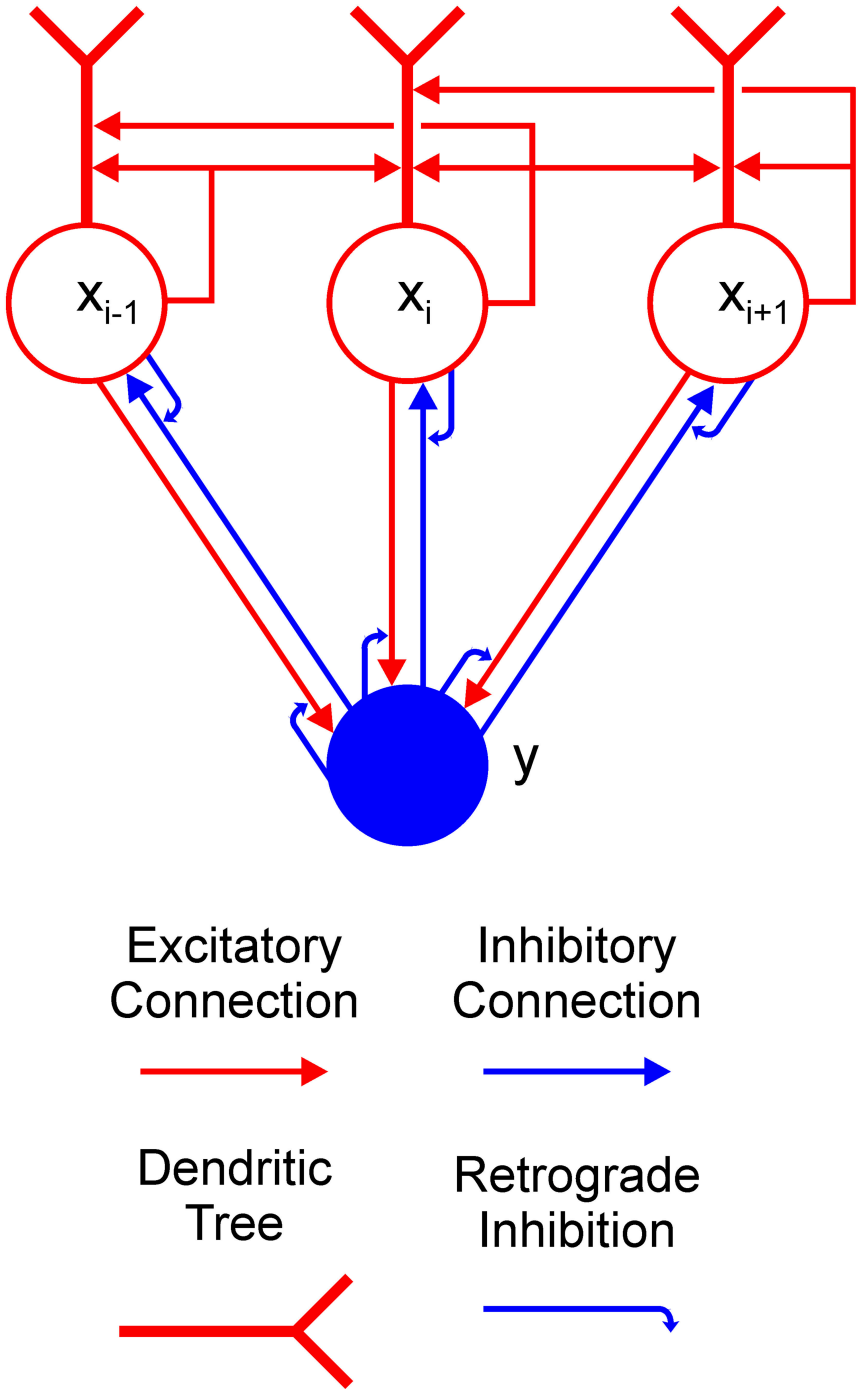
Feature-based winner-take-all (F-WTA) circuit. Connections between excitatory (red circles) and inhibitory (blue disk) units are modulated by retrograde inhibition (curved blue arrows). Self-excitation and nearest-neighbor excitation are mediated by the dendrites of the excitatory units. The same motif is repeated for all excitatory nodes in the recurrent map.

The aim of the current work is to provide an explanation of how a Boolean map may be formed in a recurrent competitive network that can implement feature-based winner-take-all (F-WTA) selection. To this end, we have extended the previously proposed network model based on the linear-threshold units (Hahnloser, 1998; Hahnloser et al., 2003; Rutishauser and Douglas, 2009). Concretely, the model circuit is presented in Figure 2. It consists of a single inhibitory unit, which is reciprocally connected to a group of excitatory units. In addition to these basic elements, we introduce two processing components into the WTA circuit to expand its computational power. The first is a dendritic non-linearity, which prevents excessive excitation that arises from self-recurrent and nearest-neighbor collaterals. We modeled the dendritic tree as a separate electrical compartment with its own non-linear output that is supplied to the node's body (Häusser and Mel, 2003; London and Häusser, 2005; Branco and Häusser, 2010; Mel, 2016). The second is modulation of synaptic transmission by retrograde inhibitory signaling (Tao and Poo, 2001; Alger, 2002; Zilberter et al., 2005; Regehr et al., 2009). This is a form of presynaptic inhibition, where postsynaptic cells release a neurotransmitter that binds to the receptors that are located on the presynaptic terminals. Retrograde signaling creates a feedback loop that dynamically regulates the amount of transmitter that is released from the presynaptic terminals. Here, we have hypothesized that such interactions occur in recurrent pathways from the excitatory nodes to the inhibitory interneuron and back from the interneuron to the excitatory nodes. In the excitatory-to-inhibitory pathway, retrograde signaling enables the inhibitory interneuron to compute the maximum instead of the sum of its inputs. Computation of the maximum arises from the limitation that the activity of the inhibitory interneuron cannot grow beyond the maximal input that it receives from the excitatory nodes. Furthermore, retrograde signaling in the inhibitory-to-excitatory pathway enables the excitatory nodes that receive maximal input to protect themselves from the common inhibition. In this way, the network can select all excitatory nodes with maximal input, irrespective of their quantity or arrangement in visual space.

At first sight, it might appear strange to propose that an excitatory unit can inhibit its input by releasing a neurotransmitter that binds to the presynaptic terminal. However, several signaling molecules have been identified to support such interactions, including endogenous cannabinoids (Alger, 2002). Moreover, Zilberter (2000) found that glutamate is released from dendrites of pyramidal neurons in the rat neocortex and suppresses the inhibition that impinges on them. In addition, similar action has been found for GABA (Zilberter et al., 1999), which suggests that conventional neurotransmitters can engage in retrograde signaling.

**Figure 3:**
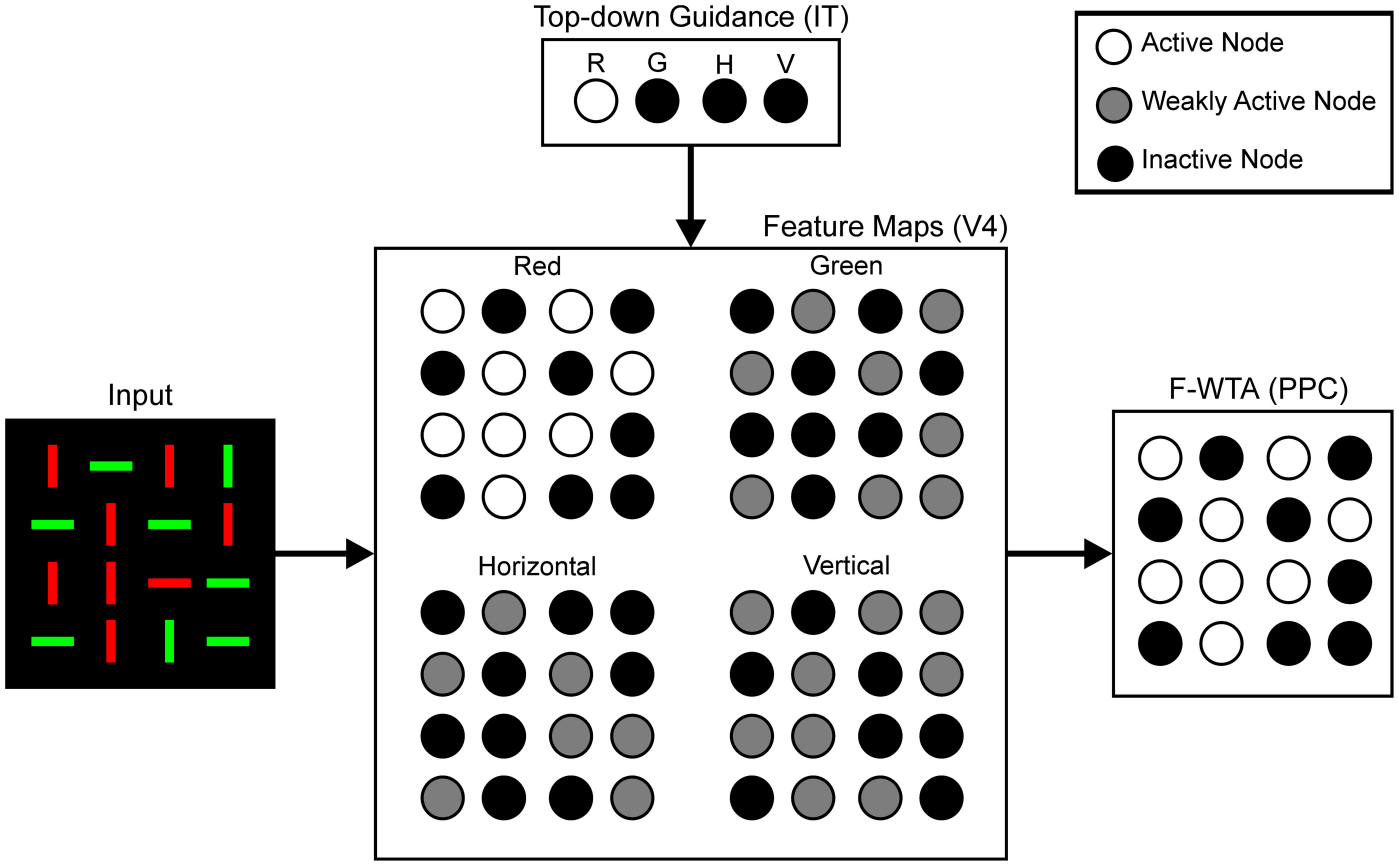
Neural architecture for the top-down guidance of attention by feature cues, following Hamker (2004). Input is processed in retinotopically organized feature maps for colors and orientations. These maps also receive top-down signals, which provide feature guidance. In this example, input image is taken from Figure 1B and red color is cued by the top-down signals. Therefore, the activity of the nodes in the Red map is enhanced (white discs) relative to the activity in all other feature maps (gray discs) because latter receives only feedforward signals. Black discs represent inactive nodes. In the feature maps, this indicates the absence of a feature at a given locations. The F-WTA network sums output of all feature maps. Its activity represents all locations occupied by the cued feature. Parentheses contain reference to cortical areas thought to be involved in proposed computations. R, red; G, green; H, horizontal; V, vertical.

To situate the proposed F-WTA circuit in a larger neural architecture that describes the cortical computations that underlie top-down attentional control, we have adopted the model that was proposed by Hamker (2004). He showed how attentional selection of a target arises from the recurrent interactions within a distributed network that consists of model cortical area V4, the inferotemporal cortex (IT), the posterior parietal cortex (PPC), and the frontal eye fields (FEF). Figure 3 illustrates part of these interactions that are involved in feature-based attentional guidance. Top-down signals that provide feature cues originate in the IT, which contains a spatially invariant representation of relevant visual features. The IT sends feature-specific feedback projections to the V4, where topographically organized feature maps for each feature value are located. For simplicity, we consider only maps for two colors (red and green), and two orientations (vertical and horizontal). We do not explicitly model IT and V4 dynamics. Rather, they serve here as a tentative explanation of how input to the F-WTA network arises within the ventral visual pathway. Also, we omitted the contribution of the FEF and its spatial reentry signals to the V4 activity.

We hypothesize that the feature-based WTA network resides in the PPC, where it receives summed input over all feature maps from the V4. Top-down guidance is implemented by a temporary increase in activity in one of the V4 feature maps. For example, when the decision is made to attend to the red color, the IT representation of red color sends feedback signals to the Red Map in the V4. Top-down signals to the feature map are modeled as a multiplicative gain of neural activity, which is consistent with neurophysiological findings (Treue and Martinez-Trujillo, 1999; Martinez-Trujillo and Treue, 2004; Maunsell and Treue, 2006).

The following neural network equations represent the quantitative description of the model. Each unit is defined by its instantaneous firing rate (Dayan and Abbott, 2000). The time evolution of the activity of excitatory node *x* at position *i* in the recurrent map is given by the following differential equation:

(1) $$\begin{array}{l} \tau_{x}\frac{dx_{i}}{dt}+x_{i}={\left[I_{i}\left(t\right)+\alpha f\left(x_{i}+x_{i+1}+x_{i-1}\right)-\beta_{1}g\left(y-x_{i}-T_{y}\right)\right]}^{+}. \end{array}$$

The time evolution of the activity of inhibitory interneuron *y* is given by

(2) $$\begin{array}{l} \tau_{y}\frac{dy}{dt}+y={\left[\beta_{2}\underset{i}{\sum }g\left(x_{i}-y-T_{x}\right)\right]}^{+}. \end{array}$$

Parameters τ_*x*_ and τ_*y*_ are integration time constants for excitatory and inhibitory nodes, respectively. We assume that inequality τ_*x*_ > τ_*y*_ holds, which accords with the observation in electrophysiological measurements that inhibitory cells exhibit faster dynamics than excitatory cells (McCormick et al., 1985). The second term on the left-hand side of Equations (1) and (2) describes the passive decay that drives the unit's activity to the resting state in the absence of external input. Firing rate activation function [*u*]^+^ is a non-saturating rectification nonlinearity, which is defined by

(3) $$\begin{array}{l} {\left[u\right]}^{+}=\max\left(u,0\right). \end{array}$$

Following Hamker (2004), we assume that feedforward input *I*_*i*_ at time *t* to the excitatory node *x*_*i*_ in the F-WTA network is given by the sum over activity in all V4 feature maps $I_{i}^{\left(m\right)}$,

(4) $$\begin{array}{l} I_{i}\left(t\right)=\underset{m}{\sum }I_{i}^{\left(m\right)}G^{\left(m\right)}\left(t\right). \end{array}$$

In Equation (4), *m* denotes available feature maps with *m* ∈ {*red, green*} in the simulation that is reported in section Simulation of the Formation of a Single Boolean Map and *m* ∈ {*red, green, horizontal, vertical*} in the simulation that is reported in section Simulation of the Intersection and Union of Two Boolean Maps. Parameter *G*^*m*^ refers to the feature-specific, global multiplicative gain that all units $I_{i}^{\left(m\right)}$ within the same feature map *m* receive via top-down projections. As shown in Figure 2, these projections arrive from the feature representation in the IT. Multiplicative gating is generally consistent with previous models that describe the effect of feature-based attention on the responses of neurons in the early visual cortex (Boynton, 2005, 2009). Equation (4) ensures that the F-WTA network is not particularly sensitive to any feature value. Rather, it signals the behavioral relevance of locations in a spatial map. Here, the relevance can be set according to differences in the bottom-up input $I_{i}^{\left(m\right)}$ that arise from competitive interactions in the early visual cortex. Alternatively, relevance can be signaled by the top-down feature cues *G*^*m*^ that change the gain of all locations that are occupied by the same feature value.

Dendritic output *f(u)* is described by the sigmoid response function

(5) $$\begin{array}{l} f\left(u\right)=\frac{S_{d}}{1+e^{-\lambda \left(u-T_{d}\right)}} \end{array}$$

where λ and *T*_*d*_ control the shape of the sigmoid function and *S*_*d*_ is its upper asymptotic value. We set λ to a high value to achieve a steep rise of the dendritic activity immediately after its input crosses the dendritic threshold, which is denoted as *T*_*d*_. Such strong non-linearity is justified by experimental data, which show all-or-none behavior in real dendrites (Wei et al., 2001; Polsky et al., 2004). In Equation (1), parameter α controls the strength of the impact that the dendritic compartment exerts on the soma.

Self-recurrent *x*_*i*_ and nearest-neighbor collaterals *x*_*i*−1_ and *x*_*i*+1_ arrive on the dendrite of the excitatory node, which is consistent with the anatomical observation that most recurrent excitatory connections are made on the dendrites of the excitatory cells (Spruston, 2008). Nodes at the edge of the network receive excitation only from a single available neighbor. That is, node *x*_1_ receives excitation only from *x*_2_, and *x*_*N*_ receives excitation only from *x*_*N*−1_. Nearest-neighbor excitatory interactions enable feature cues to spread activity enhancement automatically to all connected locations that contain a given feature value. This is not essential for the simulation of Boolean maps but we included it in our model because recurrent connections among nearby neurons are prominent feature of the synaptic organization of the cortex (Douglas and Martin, 2004). Also, we wanted to show that the proposed model is capable of simulating object-based attention (Roelfsema, 2006; Roelfsema and de Lange, 2016). Moreover, Wannig et al. (2011) found direct evidence for activity spreading among neurons that encode the same feature value in the primary visual cortex.

The output of the presynaptic interactions *g(u)* is defined by the rectification non-linearity of the form

(6) $$\begin{array}{l} g\left(u\right)={\left[u\right]}^{+}=\max\left(u,0\right). \end{array}$$

**Figure 4:**
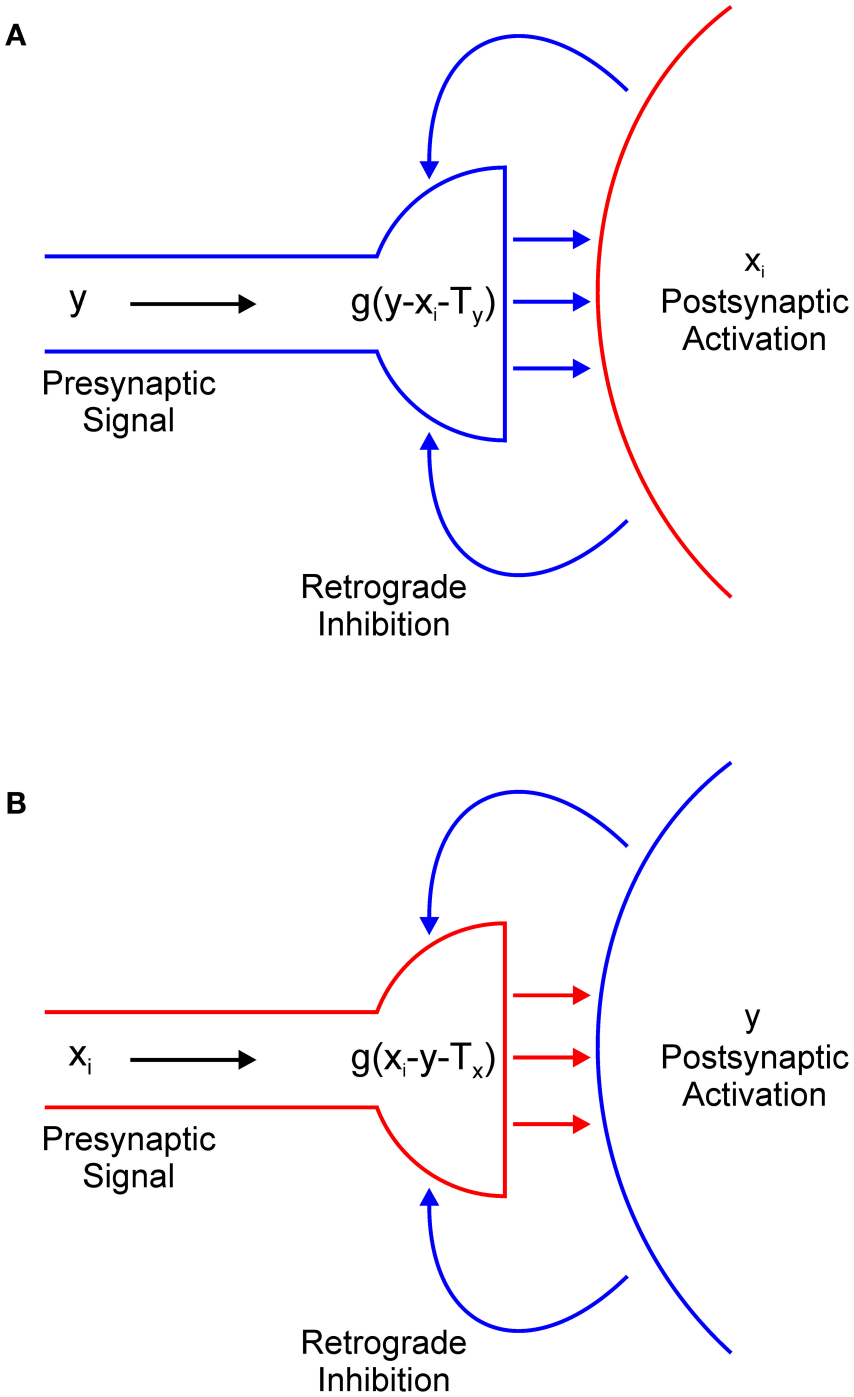
Retrograde inhibitory signaling (blue curved arrows) from excitatory node *x*_*i*_ to the presynaptic terminal of inhibitory interneuron *y* **(A)** and from the inhibitory interneuron to the presynaptic terminal of the excitatory node **(B)**. Both terminals compute half-wave rectification *g*(*u*) of their input. Terminals release respective inhibitory **(A)** or excitatory **(B)** neurotransmitter (straight horizontal arrows) only when they receive net positive input.

In Equation (1), the term − *g*(*y* − *x*_*i*_ − *T*_*y*_) describes the output of the presynaptic terminal that delivers inhibition from interneuron *y* to excitatory node *x*_*i*_ (Figure 4A). However, we did not explicitly model the dynamics of retrograde signaling. We assumed that the release of the retrograde transmitter occurs simultaneously with the activation of the postsynaptic node and that it is proportional to its firing rate. Therefore, it is represented by the term − *x*_*i*_.

Function *g*(*u*) ensures that the presynaptic terminal will release the inhibitory transmitter only when the electrical signal from node *y* exceeds the inhibitory retrograde signal *-x*_*i*_ and the threshold for presynaptic activation, which is denoted as *T*_*y*_. In other words, node *x*_*i*_ will be inhibited only if *y* > *x*_*i*_ + *T*_*x*_. If this is not the case, node *x*_*i*_ will effectively isolate itself from the inhibitory influence of node *y*. This is always the case for the winning node because *x*(*t*) > *y*(*t*) for *t* > 0. Moreover, this result extends to all other nodes whose input magnitude is sufficiently close to the maximal input. The strength of the inhibition is determined by parameter β_1_. In a similar vein, in Equation (2), the term −*g*(*x*_*i*_ – *y* – *T*_*x*_) describes the action of the retrograde signal that is released from inhibitory interneuron *y* on the presynaptic terminal that delivers excitation from node *x*_*i*_ (Figure 4B). Here, parameter *T*_*x*_ describes the threshold for the activation of the presynaptic terminal of the excitatory node and β_2_ determines the strength of the excitation.

We have proposed a model of a one-dimensional network, although it attempts to simulate phenomena that occur in 2-D, as illustrated by Figure 1. We have chosen to work with the 1-D version of the network simply because we want to focus on the analysis of its temporal dynamics and its ability to combine information over time. Without loss of generality, the computer simulations that are reported in section Computer Simulations should be considered as a cross-section of a 2-D network.

For simplicity, the thresholds that control the activation of the excitatory and inhibitory nodes are all set to zero and are omitted from the model description. Parameters were set as follows: τ_*x*_ = 5; τ_*y*_ = 2; α = 1; β_1_ = 1; β_2_ = 10; *S*_*d*_ = 1; λ = 100; *T*_*d*_ = 0.1; *T*_*x*_ = 0.1; and *T*_*y*_ = 0.1. Parameters were chosen in a way to simultaneously achieve intersection and union. Systematic variations on the parameters α, β_1_ and β_2_ showed that intersection is observed when 1 ≤ (α, β_1_) ≤ 5. In contrast, union is observed when 0.8 ≤ (α, β_1_) ≤ 1. Parameter β_2_ can be set to any value above the default without changing the results.

## Model extensions

The network that is defined by Equations (1) and (2) is chosen in a way that achieves the desired behavior with the minimal number of computational elements. This simplicity heuristic is important for understanding model properties without adding extra neuroscientific complexity (Ashby and Hélie, 2011). However, at the same time, this approach sacrifices anatomical and biophysical plausibility of the proposed model. In this section, we present several extensions and generalizations of the basic model that bring it closer to satisfying the neurobiological constraints.

### Inhibitory pool

**Figure 5:**
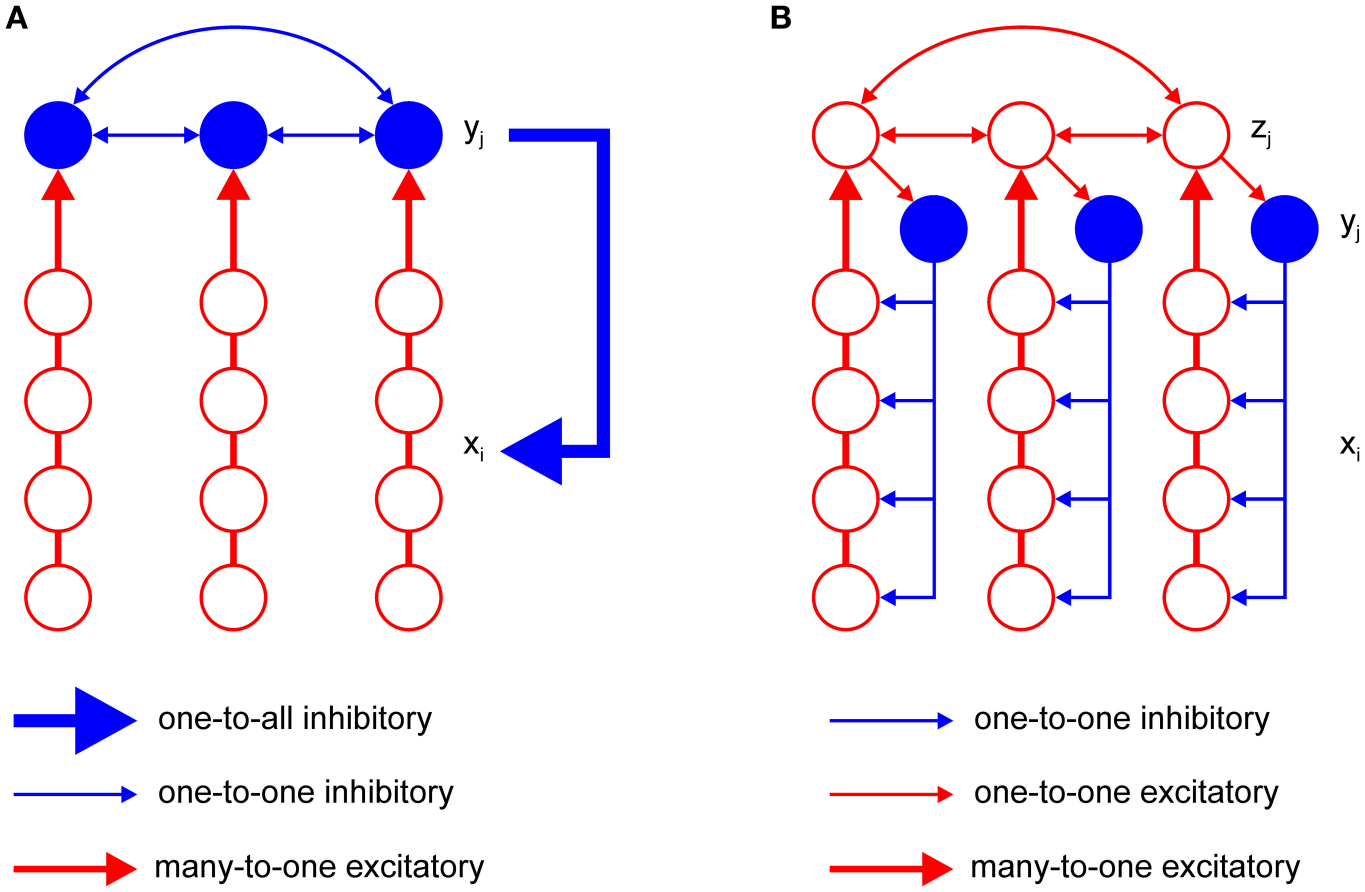
Two variations of the F-WTA circuit design that are computationally equivalent to the basic circuit that is shown in Figure 2. **(A)** Circuit with a set of inhibitory nodes, which are denoted as *y*_*j*_. Each *y*_*j*_ receives input from a subset of excitatory nodes. Inhibitory nodes compete with one another and the winning node encodes the maximum of its input. It delivers inhibition to all excitatory nodes in the same way as single inhibitory node *y* in the basic circuit. **(B)** Circuit with an additional set of excitatory nodes *z*_*j*_ with long-range horizontal projections. These nodes propagate the locally computed maximum level of activity to all parts of the network. Therefore, the whole set of *z*_*j*_ converges to a global maximum. Furthermore, they contact inhibitory nodes *y*_*j*_ that deliver inhibition to a subset of excitatory nodes *x*_*i*_.

The model has just one inhibitory interneuron for computational convenience, which is not realistic. It is known that excitatory neurons outnumber inhibitory neurons by a factor of four in the cortex (Braitenberg and Schüz, 1991). However, it is possible to design an F-WTA network with a pool of inhibitory interneurons and the appropriate ratio between excitatory and inhibitory nodes that achieves the same behavior as the original model. An extended F-WTA network is presented in Figure 5A. Here, each inhibitory interneuron receives input from a subset of the excitatory nodes. We depicted each excitatory subset as a vertical arrangement of four nodes that do not overlap in their projections to the inhibitory pool. Therefore, each excitatory node projects to just one inhibitory node. Naturally, this does not need to be the case. It is possible that each excitatory node projects to more than one node without compromising the network output. Importantly, all inhibitory interneurons are mutually connected. In addition, each inhibitory interneuron projects its output to all excitatory nodes (denoted by thick blue arrow). As in the original model, we assume that all inhibitory and excitatory nodes are endowed with the capability of retrograde signaling on their synaptic contacts.

Within the pool of inhibitory nodes, retrograde signaling enables computation of the MAX function, as in the original model. To see this, consider the inhibitory node that receives maximal input. Due to the retrograde signaling, it will reach a steady state that corresponds to the computation of the MAX function over input from its excitatory subset. Moreover, it will not receive inhibition from the other members of the pool. All other inhibitory nodes, which receive less excitatory support, will be silenced because their retrograde signaling is not sufficiently strong to prevent lateral inhibition from the winning node. However, if there are multiple inhibitory nodes with the same level of activity, they will remain active together. Finally, the winning nodes send inhibition to all excitatory subsets. Since excitatory nodes also engage in retrograde signaling, the nodes that receive maximal input will block inhibition and remain active. Therefore, the network output will look much like the original model because the MAX computation on the inhibitory nodes makes irrelevant the number of them that are active simultaneously.

### Localized inhibition

An important shortcoming of the previous model is that it assumes that inhibitory projections extend across the whole network of excitatory units. This is clearly not the case in real neural networks, where the spatial spread of inhibition is limited. To account for this property, we have constructed a more elaborate version of the basic model, which is shown in Figure 5B. It contains a new pool *z*_*j*_ of excitatory nodes with long-range projections. The *z*_*j*_ nodes receive input from the subset of the *x*_*i*_ nodes. Additionally, each *z*_*j*_ node sends its projection to at least one *y*_*j*_ node from the pool of inhibitory nodes. The number of *z* nodes must equal the number of inhibitory nodes *y*_*j*_ so that they can be indexed by the same subscript *j*. Again, we assume that the *z*_*j*_ nodes are equipped with the ability of retrograde signaling on their synapses. Therefore, they also compute the MAX function over all their inputs, including feedforward input from the corresponding subset of *x*_*i*_ nodes and recurrent input from other *z*_*j*_ nodes. In this design, the maximum level of activity that is sensed by the *x*_*i*_ nodes in one part of the network is easily propagated via *z*_*j*_ nodes to all other parts of the network. Furthermore, *z*_*j*_ nodes transfer this activity to inhibitory nodes. Therefore, each inhibitory node will eventually receive the maximal level of activity and apply it to the subset of *x*_*i*_ nodes to which it is connected. In this design, it is not necessary for inhibitory nodes to interact with one another. The excitatory nodes *x*_*i*_ that receive maximal input will block inhibition by their retrograde signaling and remain active in the same manner as described in the previous section. In this way, the proposed circuit achieves the same result as the original model.

### Output functions

The model employs threshold-linear output functions for the soma and the logistic sigmoid function for dendrites. This is inconsistent with the observation that somatic output also saturates and is also often modeled by the sigmoid function. However, in normal circumstances, neurons operate in a linear mode that is far from their saturation level (Rutishauser and Douglas, 2009). To provide a more systematic approach to the output functions that are used in the model, we introduce a piecewise-linear approximation to the sigmoid function *s*_*q*_(*u*) of the form

(8) $$s_{q}\left(u\right)=\{\begin{array}{ccc} 0 & if & u\leq 0\\ u & if & 0<u<S_{q}\\ S_{q} & if & u\geq S_{q} \end{array}$$

where *S*_*q*_ denotes the upper saturation point, which can be set differently for different computational units *q* ∈ {*c, d, p*}, which correspond to the somatic, dendritic, and presynaptic terminal outputs, respectively. With the output function *s*_*q*_(*u*) applied to all computational elements of a single node, the model equations, namely, Equations (1) and (2), can be restated as

(9) $$\begin{array}{l} \tau_{x}\frac{dx_{i}}{dt}+x_{i}=s_{c}[I_{i}\left(t\right)+\alpha s_{d}\left(x_{i}+x_{i+1}+x_{i-1}-T_{d}\right)\\ \;-\beta_{1}s_{p}\left(y-x_{i}-T_{y}\right)] \end{array}$$

and

(9) $$\tau_{y}\frac{dy}{dt}+y=s_{c}\left[\beta_{2}\sum_{i}s_{p}\left(x_{i}-y-T_{x}\right)\right].$$

An important constraint of the model that is defined by Equations (8) and (9) is that saturation point for the dendritic output *S*_*d*_ should be chosen to be smaller than *S*_*c*_, which is the saturation point of the somatic output. In this way, feedforward input *I*_*i*_ can be combined with the dendritic output without causing saturation at the output of the node. In contrast, if dendrites are allowed to saturate at the same activity level as the node, the dendritic output will overshadow the feedforward input. Consequently, the network will lose its sensitivity to the input changes. This is undesirable with respect to the requirements that are imposed by the sequential formation of the multiple Boolean maps. Therefore, the choice between the linear or the sigmoid output function for the node is not important if the dendritic output is restricted to a smaller interval relative to the output of the node itself.

## Linear stability analysis

### Fixed points

Fixed point is found iteratively starting from the set of nodes receiving maximal input, *x*_*M*_. We assume that the winning nodes and inhibitory interneuron are activated above their thresholds, so we set [*u*]^+^ = *u*. Next, we observe that the winning nodes do not receive inhibition from the interneuron *y* since *x*_*M*_*(t)* > *y(t)* for *t* > 0. This holds because the activity of the inhibitory node is bounded above by *x*_*M*_ + *T*_*x*_ > *y* where *T*_*x*_ is a positive constant. Then, retrograde signaling ensures that *g(y* − *x*_*M*_ − *T*_*y*_*)* = 0 for all times *t*. Consequently, nodes receiving maximal input are driven solely by excitatory terms. Since the recurrent excitation is bounded above by its asymptotic value *S*_*d*_, dendritic output function *f(u)* in Equation (1) is replaced with *S*_*d*_. This yields the following approximation to the steady state of the winning nodes:

(10) $$\begin{array}{l} x_{M}\approx I_{M}+\alpha S_{d}. \end{array}$$

After the *x*_*M*_, inhibitory interneuron *y* also reaches its steady state because its activity is driven primarily by the input from *x*_*M*_. As the activity of *y* grows, terms *g(x*_*i*_ − *y* − *T*_*x*_*)* in Equation (2) vanish for all nodes that do not receive maximal input *x*_*i*_ where *i* ∉ *M*. In contrast, the presynaptic terminals of *x*_*M*_ are above the threshold for their activation just before *y* reaches equilibrium, that is, *g(x*_*M*_ − *y* − *T*_*x*_*)* > *0*. Therefore, the output function of the presynaptic terminal *g(u)* can be replaced by *u*. Then, Equation (2) is solved as

(11) $$\begin{array}{l} y=\frac{\beta_{2}k\left(x_{M}-T_{x}\right)}{\beta_{2}k+1}, \end{array}$$

where *k* is the number of *x*_*M*_. When β_2_ is chosen to be sufficiently large, and/or there are many nodes with maximal input *x*_*M*_, then

(12) $$\begin{array}{l} y\to x_{M}-T_{x}. \end{array}$$

Continuity of the function defined by Equation (2) implies that *y* cannot grow above *x*_*M*_ − *T*_*x*_, that is, *y(t)* > *x*_*M*_*(t)* − *T*_*x*_ cannot hold at any time *t* unless *y(t*_0_*)* = *x*_*M*_*(t*_0_*)* − *T*_*x*_ at some earlier time *t*_0_ < *t*. However, equality *y(t*_0_*)* = *x*_*M*_*(t*_0_*)* − *T*_*x*_ implies that *dy/dt* = *0* at time *t*_0_ because *g(x*_*M*_*(t*_0_*)* − *y(t*_0_*)* − *T*_*x*_*)* = *0*. In other words, node *y* loses all its excitatory drive when it reaches *x*_*M*_ − *T*_*x*_. This is true irrespective of the number *k* of *x*_*M*_. Thus, node *y* computes the maximum over its input.

The *x*_*M*_ nodes, together with the inhibitory node, create a quenching threshold (QT) for the network, which is defined by

(13) $$\begin{array}{l} QT=y-T_{y}=x_{M}-T_{x}-T_{y}. \end{array}$$

Grossberg (1973) introduced the concept of the quenching threshold to describe the property of contrast enhancement in recurrent competitive networks. Nodes whose activity is above QT are enhanced and stored in the memory state, while all nodes whose activity is below QT are suppressed and removed from the memory representation. In the same manner, the remaining excitatory nodes converge to one of two states, depending on whether they exceed QT or not:

(14) $$x_{i\notin M}\approx \{\begin{array}{ccc} I_{i}+\alpha S_{d} & if & x_{i}\geq QT\\ 0 & if & x_{i}<QT. \end{array}$$

**Figure 6:**
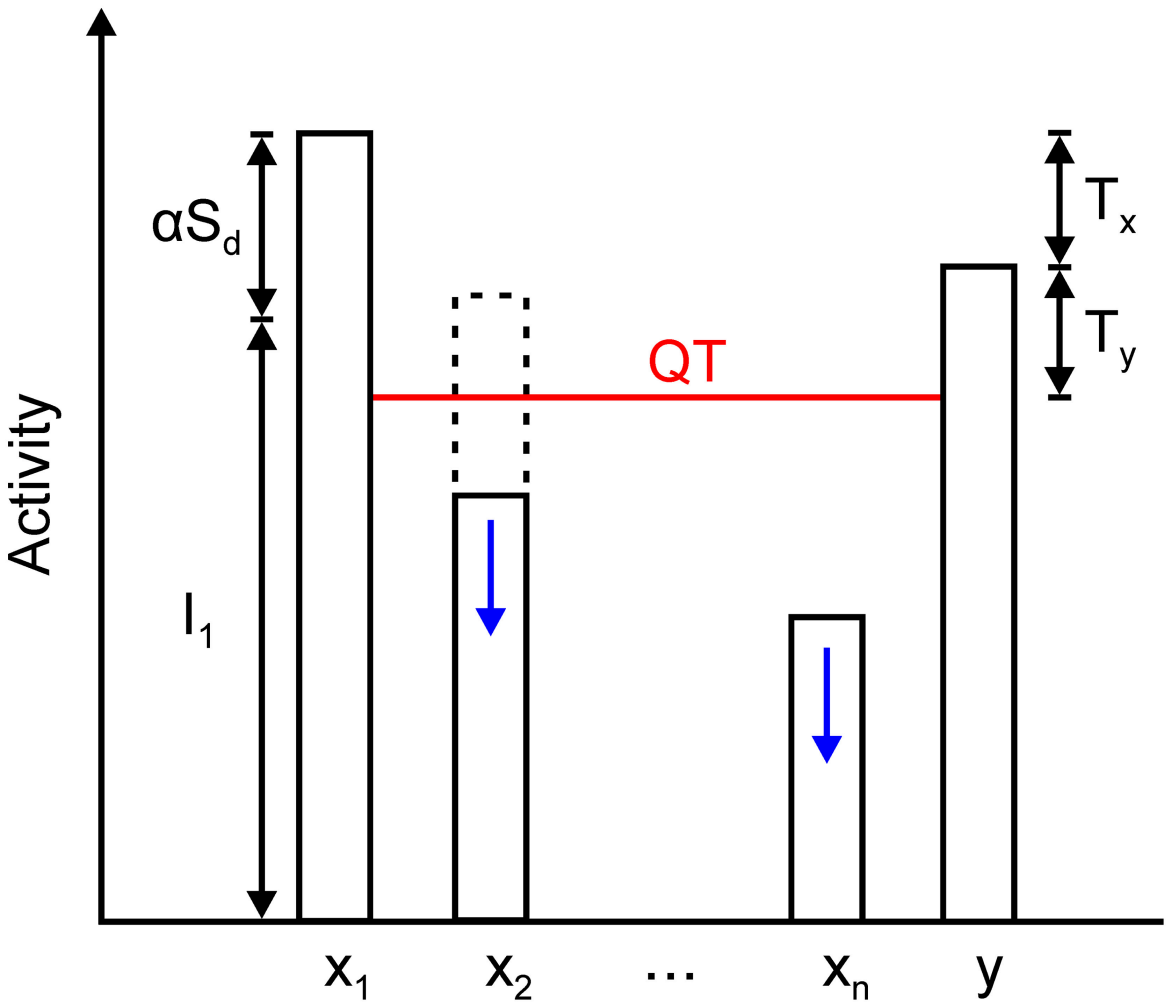
Relationship among the steady state of the winning node *x*_1_, inhibitory node *y*, and all other excitatory nodes in the network, *x*_2_ … *x*_*n*_. The activity of the winning node is given by the sum of its feedforward input *I*_1_ and the output of its dendrite mediating self- and nearest neighbor excitation, which is expressed as α*S*_*d*_. Inhibitory node y approximately converges to *x*_1_ – *T*_*x*_. It sets the quenching threshold (QT) that separates excitatory nodes into two sets. Nodes *x*_2_ … *x*_*n*_ are spared from inhibition if their activity is above the QT (dashed line); otherwise, they are silenced to zero (solid line). QT equals *y* – *T*_*y*_ (or *x*_1_ – *T*_*x*_ – *T*_*y*_) because the activity of the inhibitory node must exceed the threshold on its presynaptic terminals that contact the excitatory nodes.

QT and its relationship with the activity of the winning and non-winning nodes and inhibitory interneuron is illustrated in Figure 6. According to Equations (10), (11), and (14), the fixed-point linearly combines input and recurrent excitation. As maximal input increases or decreases, the fixed point will move up or down and track these changes. Moreover, the input may cease, and the winning nodes will settle into the activity level that is provided by the recurrent excitation alone, which is expressed as α*S*_*d*_. In other words, the network remembers who the last winner was. The same is true in the case where the winner is determined by transient cues that are applied sequentially on a sustained input. This is a protocol that is used in the computer simulations that are reported in section Computer Simulations.

### Linearization near fixed points

To simplify the stability analysis, we consider an F-WTA network with two excitatory nodes and one inhibitory node: [*x*_1_, *x*_2_, *y*]. This system has three fixed points: *x*_1_ is the only winner, *x*_2_ is the only winner, and both excitatory nodes are winners. To which fixed point the network will converge depends on the relationship between inputs *I*_1_ and *I*_2_.

Local stability of the fixed point is estimated from the eigenvalues of the Jacobian matrix, which is the matrix of partial derivatives of the system of equations. If the real parts of all eigenvalues of the Jacobian are negative, the fixed point will be asymptotically stable (Rutishauser and Douglas, 2009). However, before we can compute the Jacobian matrix, we note that a linear-threshold function is continuous, but not differentiable. To sidestep this problem, we follow the approach that was described by Rutishauser et al. (2011) of inserting dummy terms that correspond to the derivate. That is, we need three separate dummy terms: *c*_*i*_ and *p*_*xi*_, which correspond to the somatic and presynaptic output functions of excitatory node *i*, and a set of *p*_*yi*_ dummy terms that describe the presynaptic output function of inhibitory node *y*. The dummy terms are defined as.

(15) $$c_{i}=p_{xi}=p_{yi}=\frac{d}{du}{\left[u_{i}\left(t\right)\right]}^{+}=\{\begin{array}{ccc} 0 & if & u_{i}\left(t\right)\leq 0\\ 1 & if & u_{i}\left(t\right)>0. \end{array}$$

Based on the above definition of the dummy terms, we have constructed the Jacobian matrix of the system that consists of Equations (1) and (2):

(16) $$\begin{array}{l} J=\left[\begin{array}{ccc} \tau_{x}^{-1}\left(c_{1}\left(\alpha D_{1}f+\beta_{1}p_{y1}\right)-1\right) & \tau_{x}^{-1}c_{1}\alpha D_{2}f & \tau_{x}^{-1}c_{1}\beta_{1}p_{y1}\\ \tau_{x}^{-1}c_{2}\alpha D_{1}f & \tau_{x}^{-1}\left(c_{2}\left(\alpha D_{2}f+\beta_{1}p_{y2}\right)-1\right) & \tau_{x}^{-1}c_{2}\beta_{1}p_{y2}\\ \tau_{y}^{-1}\beta_{2}p_{x1} & \tau_{y}^{-1}\beta_{2}p_{x2} & -\tau_{y}^{-1}\left(\beta_{2}\left(p_{x1}+p_{x2}\right)-1\right) \end{array}\right] \end{array}$$

where *D*_1_*f* and *D*_2_*f* denote the partial derivatives of the sigmoid function with respect to *x*_1_ and *x*_2_. Now, we examine the Jacobian matrix at the three fixed points that are mentioned above. If *x*_1_ is the only winner, then *c*_1_ = 1. However, *D*_*x*1_*f* ≈ 0 because the recurrent excitation of the winning node approaches its asymptotic value, which is *S*_*d*_. In addition, *p*_*y*1_ = 0 because the winning node blocks inhibition from node *y*, as discussed above. Node *x*_2_ is inhibited below its somatic threshold, that is, *c*_2_ = 0. Presynaptic signaling by inhibitory node *y* blocks excitation from *x*_1_ and *x*_2_ is inactive, so *p*_*x*1_ = *p*_*x*2_ = 0. Consequently, the Jacobian matrix at the fixed point reduces to a diagonal matrix of the form

(17) $$\begin{array}{l} J_{W1}=J_{W2}=J_{W12}=\left[\begin{array}{ccc} -\tau_{x}^{-1} & 0 & 0\\ 0 & -\tau_{x}^{-1} & 0\\ 0 & 0 & -\tau_{y}^{-1} \end{array}\right]. \end{array}$$

All eigenvalues of the *J*_*W*1_ are negative, and the fixed point is asymptotically stable. In the case when *x*_2_ is the sole winner, the same arguments are applied to set the dummy terms, thereby leading to the same diagonal matrix *J*_*W*2_ as shown in Equation (17). Moreover, if both excitatory nodes are winners, then *c*_1_ = *c*_2_ = 1, *D*_*x*1_*f* = *D*_*x*2_*f* ≈ 0 and *p*_*x*1_ = *p*_*x*2_ = 0. Again, the Jacobian matrix *J*_*W*12_ is diagonal. Thus, all three fixed points are asymptotically stable.

The same analysis can be generalized to a network of arbitrary size and arbitrarily many fixed points. Retrograde signaling and dendritic saturation will ensure that the Jacobian matrix of any size will be diagonal and that the network dynamics will be independent of the network parameters, namely, α, β_1_, and β_2_. Local stability analysis suggests that the system behaves much like a feedforward network that is driven by the input. However, an important difference is that the F-WTA network has memory states like the recurrent network (Usher and Cohen, 1999; Rutishauser and Douglas, 2009).

## Computer simulations

We performed a set of computer simulations to illustrate the model behavior. We employed a vector of 200 excitatory units and one inhibitory unit. Differential Equations (1) and (2) were solved numerically using MATLAB's *ode15s* solver. The simulations were run for 250 time steps. In subsequent figures, we followed the convention that activity of the node at position *i* as a function of time is depicted by a shade of gray, with white representing the maximal value and black representing zero.

### Simulation of the formation of a single boolean map

**Figure 7:**
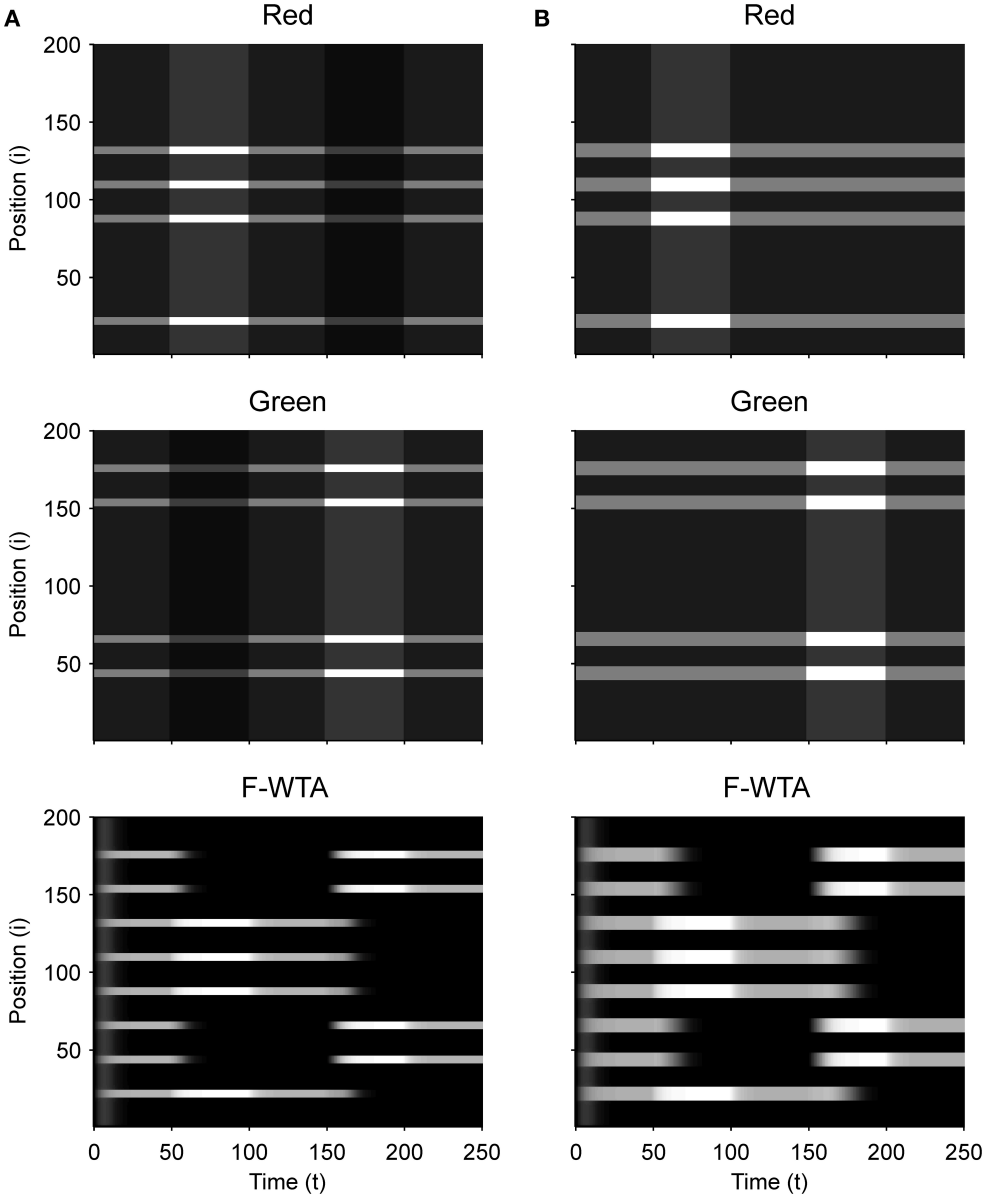
**(A)** Simulation of the Boolean map formation in the F-WTA network in response to the sequential presentation of two color cues (red appeared between 50th and 100th and green appeared between 150th and 200th simulated time unit). **(B)** The same simulation with larger items and without gain modulation applied on the unattended feature map.

First, we demonstrate how a Boolean map arises in the F-WTA network in response to the presentation of the color cue, as illustrated by Figure 1A. In Figure 7A, we recreate a similar stimulus condition in the 1-D map. The input consists of red and green items of equal sizes, which are intermixed in space on a black background. Input magnitude *I* was set to 1 in both maps and to 0.2 in the empty space around items to represent spontaneous activity in the absence of visual stimulation. Initially, the top-down or attentional gain is set to *G*^*m*^ = 1 in both feature maps*m* ∈ {*red, green*}. At *t* = 50, the red color is attended, which is reflected in the input to the network by increasing the gain for all nodes in the Red map (*G*^*red*^ = 2) and simultaneously reducing the gain in the Green map by the same factor (*G*^*green*^ = 1/*G*^*red*^ = 1/2). Top-down gain is also applied to the empty space between items, which is consistent with the finding that feature-based attention spreads across the whole visual field (Saenz et al., 2002, 2003; Serences and Boynton, 2007). The duration of the top-down cue is 50 simulated time steps. For simplicity, top-down signals are suddenly switched on and off without exponential decay. At *t* = 150, the green color is cued in the same way.

At the beginning of the simulation, before the top-down signals are applied, the F-WTA network simply selects all presented items together, irrespective of their color. Next, when the red color is cued by applying top-down signals to the corresponding feature map, the network responds to the new input by selectively increasing and sustaining the activity of nodes that encode locations of red items in the input and suppressing locations that encode green items. That is, the network creates a Boolean map by highlighting the spatial pattern that is associated with the red color. Furthermore, due to a self-excitation, the network maintains locations of the cued feature value in working memory after the top-down signals cease to influence the feature map. When the observer decides to switch attention to another feature value, the network can select the locations of the new feature value and suppress the locations that are associated with the previously cued value without requiring an external reset. Namely, the network is sensitive to input changes even though it also exhibits activity persistence.

Importantly, the activity level at selected locations is invariant with respect to the number of active nodes. At the beginning of the simulation, the number of active nodes was four times larger than after the cue was delivered. However, the active nodes remained at the same activity level as they were at the beginning of the simulation. This is a consequence of retrograde inhibitory signaling in recurrent pathways. It prevents unbounded growth of inhibition due to the dynamic regulation of its strength. To illustrate this point further, we run another simulation with items that are almost double in size (Figure 7B). Even though the total size of the cued items is increased, the activity of the cued nodes converges to the same level as before. In this simulation, we also checked that the network successfully operates even if we remove gain reduction from the non-attended feature map.

Next, we determined the minimal feature gain that must be applied on the input to produce the desired behavior. When the gain modulation is applied simultaneously on attended feature map *G*^*A*^ and on unattended feature map *G*^*NA*^ (where *G*^*NA*^ = 1/*G*^*A*^), we found that *G*^*A*^ ≥ 1.7 is sufficient for creating a Boolean map and switching to another one. In contrast, when the gain modulation is not applied on the unattended feature map, as shown in Figure 7B, the feature gain in the attended map should be set to *G*^*A*^ ≥ 2 to achieve the same behavior.

**Figure 8:**
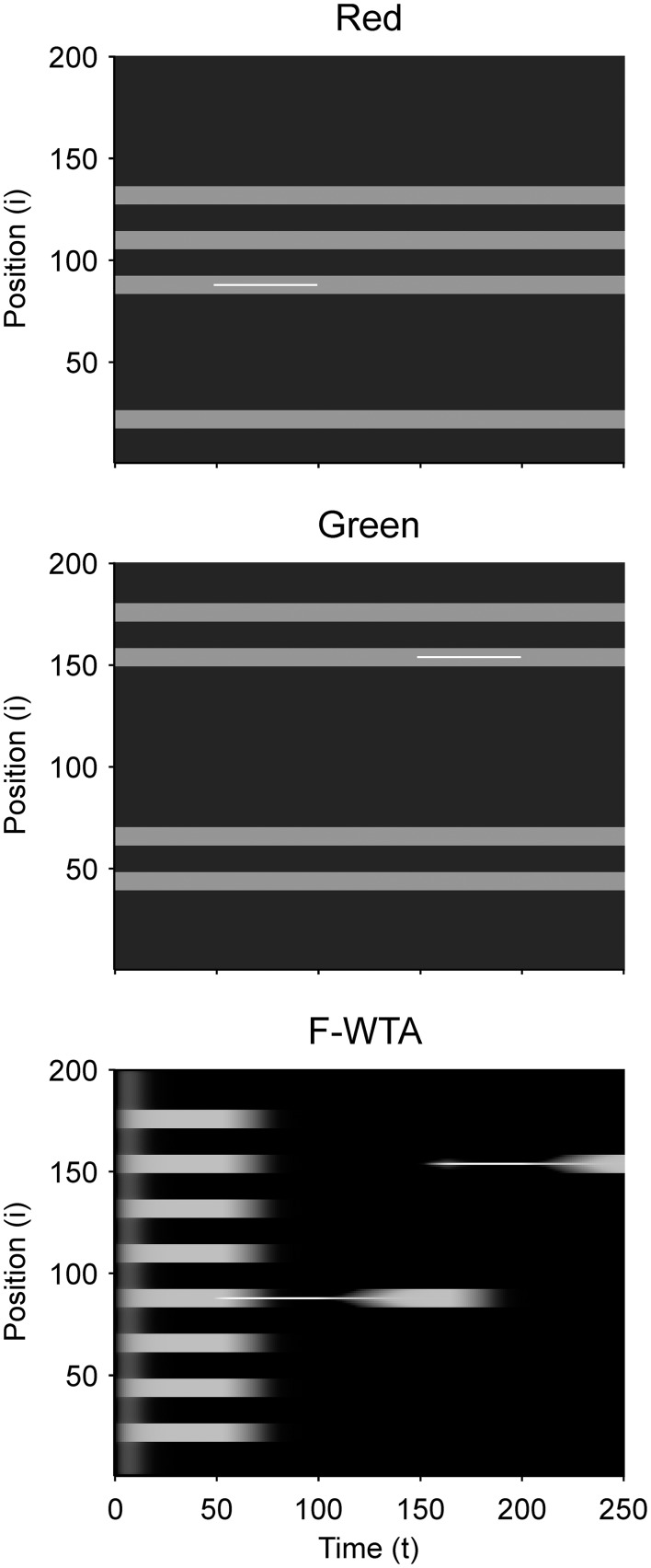
Simulation of space- and object-based attention in the F-WTA network.

Figure 8 illustrates that the F-WTA network can support space- and object-based attention alongside feature-based attention. When the spatial cue is applied to a single location in one of the feature maps, the network responds by selecting only this location. Neighboring nodes are not selected even though they are reciprocally connected to the cued node. The reason is that they receive weaker input relative to the cued node. Furthermore, recurrent excitation that arrives from the cued node is bound by the dendritic non-linearity. Thus, it is not sufficiently strong to keep them active. Interestingly, when the spatial cue is removed, the network activity starts to propagate from the cued node toward the boundary of the whole item. In this case, the network selects not just the cued location, but all locations that are connected to it. Therefore, the F-WTA network exhibits object-based selection, which is consistent with neurophysiological studies that show spreading of enhanced activity along the shape of the object (Roelfsema, 2006). This property arises because the removal of the cue equalizes the input magnitude along the object, which allows activity enhancement to propagate via local lateral connections. In addition, this simulation shows that spatial attention can be easily oriented toward a new location in a single jump without the need for attentional pointers that move attention across the map (Hahnloser et al., 1999).

### Simulation of the intersection and union of two boolean maps

**Figure 9:**
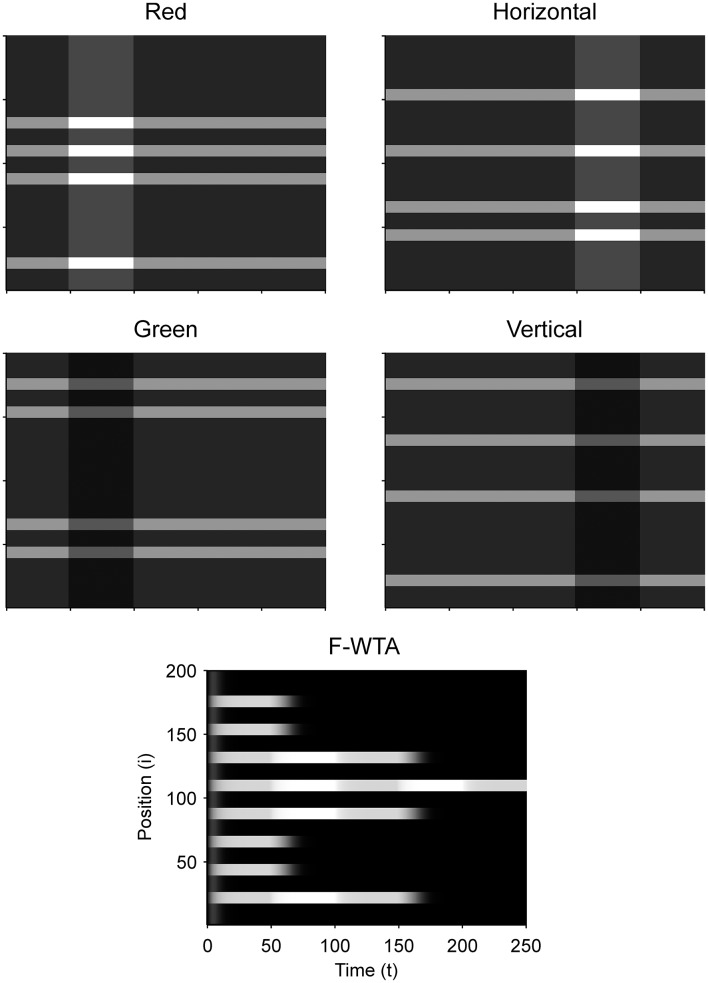
Simulation of the intersection of red and horizontal items.

Figure 9 illustrates that the model can sequentially combine two Boolean maps when the network is cued by top-down signals from two separate feature dimensions. In this simulation, we have employed a visual input that consists of red and green horizontal and red and green vertical bars, like those that are illustrated in Figure 1B. First, the F-WTA network is cued to select red bars, irrespective of their orientation. In the second step, it is cued to select horizontal bars, irrespective of their color. However, green vertical bars are already suppressed and the top-down signal that is supplied to them is not sufficient to override the inhibition that arises from red vertical bars. The net result is the selection of a subset of red horizontal bars. In other words, the network activity converges to an intersection between a set of red bars and a set of horizontal bars, thereby resulting in the selection of red horizontal bars.

**Figure 10:**
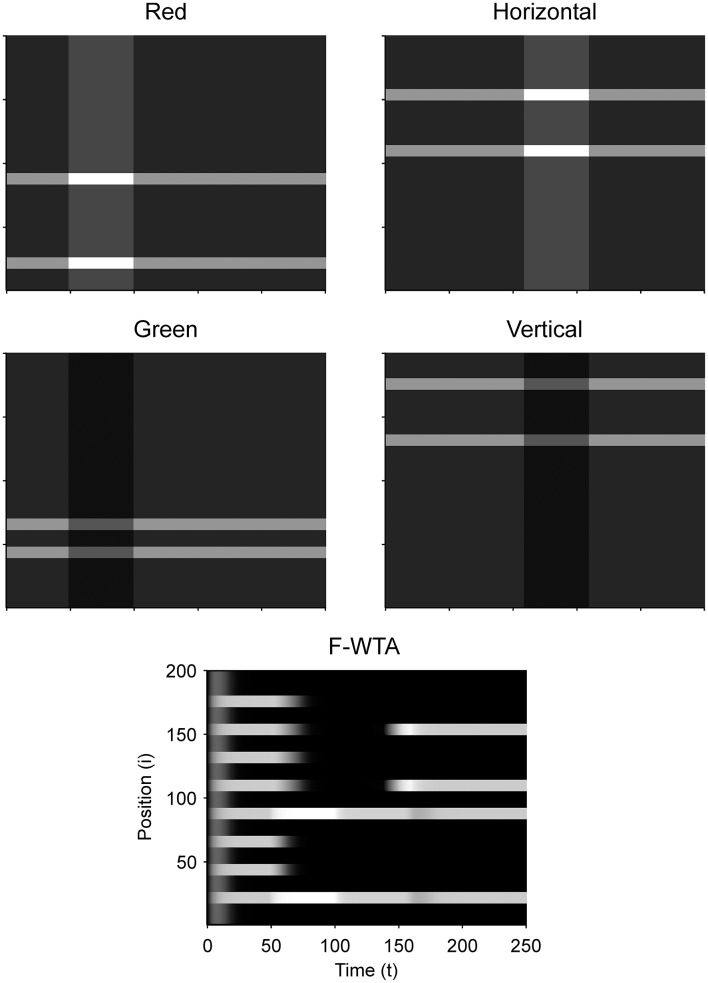
Simulation of the union of red and horizontal items.

**Figure 11:**
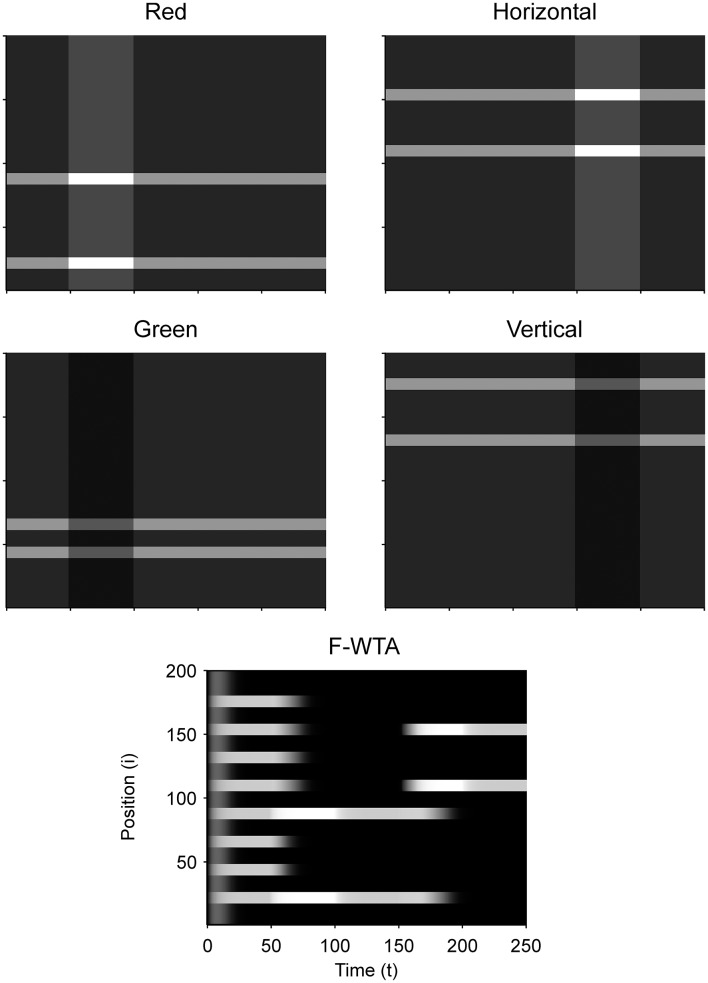
Breakdown of union of red and horizontal items when the delivery of top-down cues for red and horizontal items is separated by a large temporal gap.

Next, we examined how the network achieves the union of two Boolean maps (Figure 10). Here, we assumed that the input consists of two non-overlapping components: colored squares that activate color maps but do not activate orientation maps, and achromatic horizontal and vertical bars that activate orientation maps but do not activate color maps, as shown in Figure 1C. Red-colored items occupy locations between 1 and 100 and oriented bars occupy locations between 101 and 200. This closely resembles the stimulus that is used by Huang and Pashler (2007) to demonstrate the union of color and texture. Taken together, the data show that the union of two Boolean maps is possible only when two top-down cues overlap in time or when the second cue closely follows the withdrawal of the first cue. In Figure 10, the cue for the red map is applied in the interval [50, 100] and the cue for the horizontal map is applied in the interval [110, 160]. In this case, the F-WTA network converges to the union of red and horizontal items. However, when top-down cues do not overlap, as shown in Figure 11, the second cue overrides the network activity that remains from the first cue. We suggest that this property partly explains why the union is difficult to achieve, as observed by Huang and Pashler (2007).

In addition, we examine the boundary conditions on the choice of the feature gain parameter. We parametrically vary the feature gain in steps of 0.1 starting from *G* = 2 and moving below and above to determine when the ability to form the intersection or union breaks down. When the gain modulation is applied simultaneously on attended (*G*^*A*^) and unattended (*G*^*NA*^) feature maps, we find that *G*^*A*^ should be chosen from the interval [1.5, 2.1] to achieve the intersection between two maps. When *G*^*A*^ < 1.5, the network fails to segregate cued from non-cued locations in the first step. In contrast, when *G*^*A*^ > 2.1, the network successfully segregates cued from non-cued locations in the first step. However, the gain is too high, so all horizontal items are selected together in the second step. That is, the representation of red horizontal items is merged with the representation of green horizontal items. When *G*^*NA*^ = 1 throughout the simulation, *G*^*A*^ should be chosen from the interval [1.8, 2.0] to achieve intersection.

With respect to the union of two maps, the feature gain *G*^*A*^ should be chosen from the interval [1.4, 2.0] when *G*^*NA*^ = 1/*G*^*A*^ and from the interval [1.6, 2.0] when *G*^*NA*^ = 1. When *G*^*A*^ is chosen below the suggested intervals, feature gain is too weak, and the second cue will not be able to raise the activity level of the nodes that represent horizontal items above the quenching threshold. Therefore, the network ends up with the Boolean map of red items that is formed in the first step. When *G*^*A*^ is chosen above the suggested interval, the network switches between the representation of the red items in the first step to the representation of the horizontal items in the second step. In this case, the feature cue is too high, and the activity of the nodes that represent horizontal items simply overrides the activity of the nodes that represent the red items. These constraints are derived from the situation in which the two top-down cues overlap in time. As shown above, temporal lag of the second cue relative to the first cue also destroys the ability of the network to form the union of two Boolean maps.

### Simulation of bottom-up spatial selection

**Figure 12:**
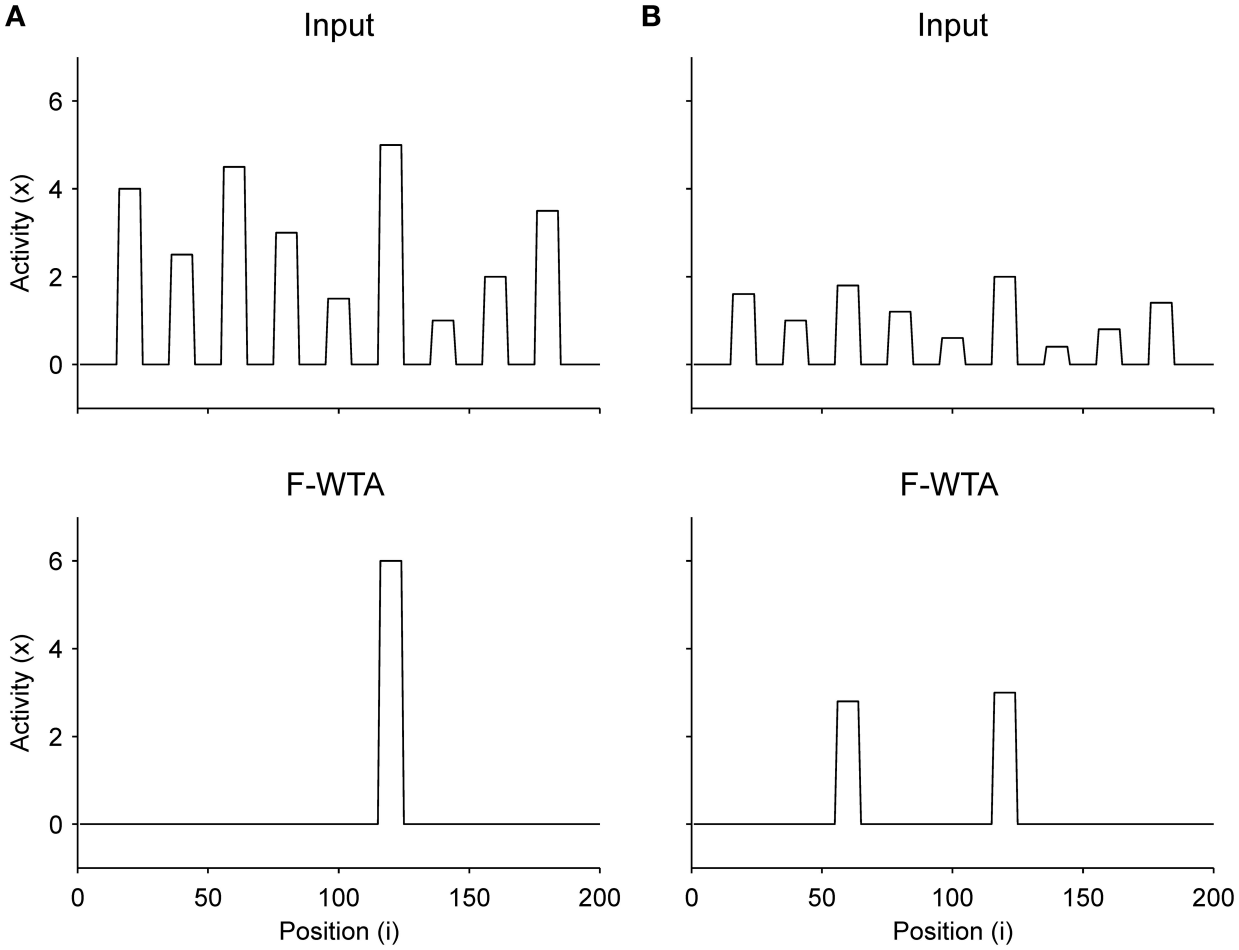
Selection of the most salient item in the absence of top-down guidance. **(A)** When the most salient item is sufficiently distinctive from other items, the F-WTA network selects it. **(B)** When the saliency of all items is relatively low, the F-WTA network may select more than one item because it has a limit on the precision by which it separates inputs of different magnitudes.

Finally, we have shown that when there is no top-down guidance, the network selects the most-salient locations based on the bottom-up salience that is computed within feature maps (Figure 12). We did not explicitly model competition among maps, but it is reasonable to assume that in a scene with many multi-featured objects, their input magnitudes (i.e., saliencies) will be different. Therefore, we arbitrarily assigned different input magnitudes to different items. As shown in Figure 12A, the F-WTA network selects the most salient object if the difference in input magnitude between the two most active nodes is sufficiently large. However, when this difference is small, as shown in Figure 12B, the F-WTA model chooses two most salient items together. Furthermore, in both examples, the network activity retains the input amplitude of the winning item (or items), thereby illustrating the ability to compute the function maximum (Yu et al., 2002).

The precision of saliency detection depends on the threshold for the activation of synaptic receptors on the inhibitory interneuron. In all reported simulations, it was set to *T*_*y*_ = 0.1. If smaller values were chosen, the network would improve in terms of precision and be able to separate the two objects that are presented in Figure 12B. However, this comes at the price of losing the ability to form a union of two Boolean maps. Therefore, there is a trade-off between the precision of saliency detection and the ability to form Boolean maps.

**Figure 13:**
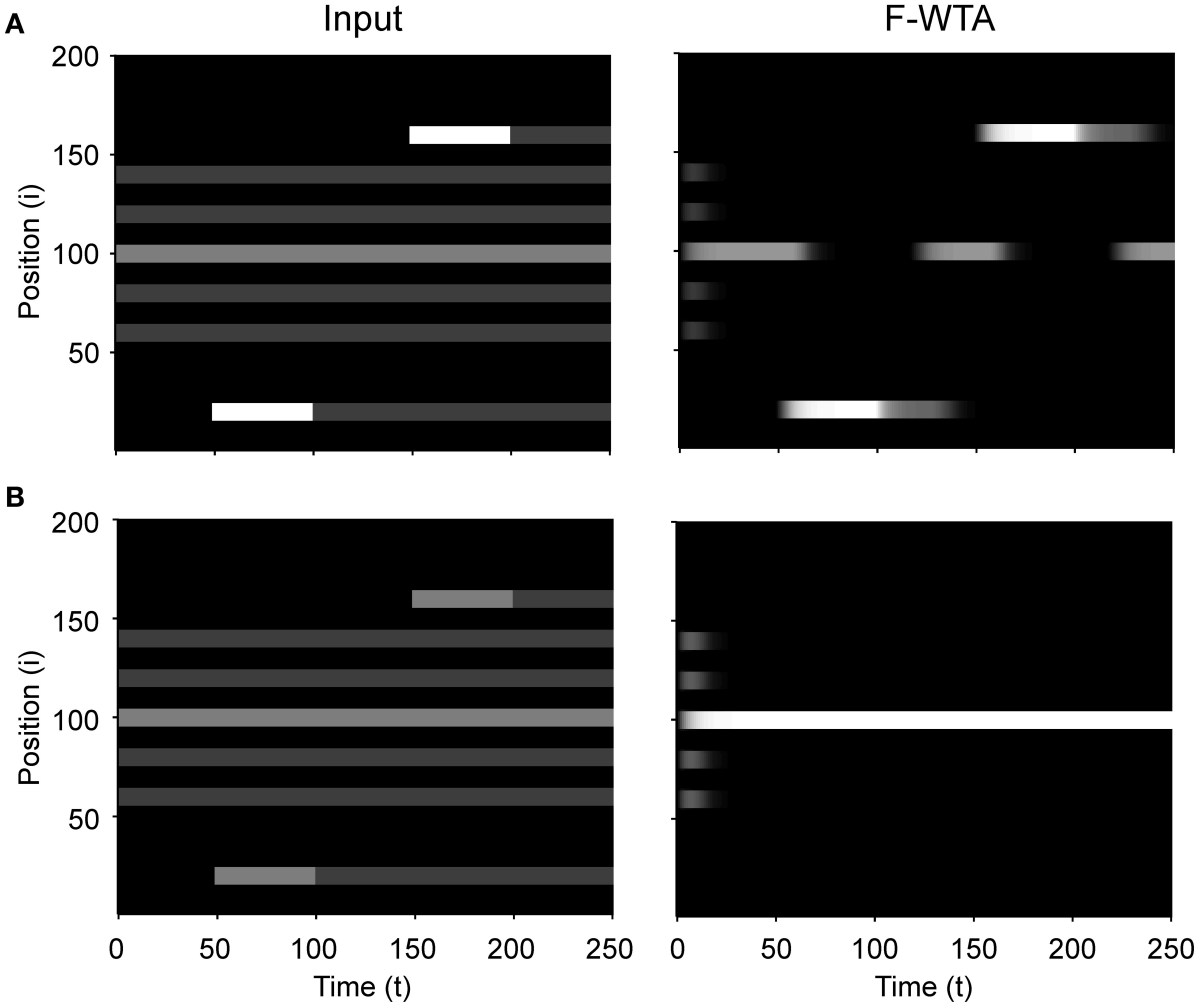
Sensitivity to abrupt visual onsets. **(A)** When the transient signal that is produced by the abrupt onset of a new object is sufficiently strong, it temporarily draws attention to itself. **(B)** When the transient signal is weak, attention resists abrupt onset and stays on the item that was selected at the beginning of the simulation.

An important aspect of stimulus-driven attentional control is attentional capture by peripheral cues. Behavioral studies have shown that the abrupt onset of a new object in a visual scene can automatically capture attention even if it is irrelevant for the current goal (Theeuwes, 2010). Figure 13 illustrates the sensitivity of the F-WTA network to abrupt visual onset. To simulate this effect, we have made the additional assumption that the network receives input not only from a sustained channel that is comprised of feature maps in V4 but also from a transient channel that responds vigorously only to changes in input (Kulikowski and Tolhurst, 1973; Legge, 1978). Thus, when the abrupt onset is accompanied by a strong transient signal that exceeds the activity level of the currently attended item, the F-WTA network temporarily switch activity toward the location of the onset (Figure 13A). Here, the input at the locations that are occupied by the winning item in the center of the map was set to *I*_*W*_ = 2. Input to all other items was set to *I*_*i*_ = 1. Finally, the transient input that appears on the sides of the map was set to *I*_*T*_ = 4. It is sufficient to set *I*_*T*_ ≥ *I*_*W*_ + 0.8 to achieve sensitivity to abrupt onsets. Moreover, the same relation holds even if we choose a larger value for *I*_*W*_.

Next, when abrupt onset produces only weak transient signals (*I*_*T*_ = 2) that do not satisfy the inequality that is stated above (*I*_*W*_ = 2), the activity in the F-WTA network resists abrupt onset and stays on the previously attended item (Figure 13B). This observation is consistent with behavioral findings that abrupt onset can be ignored (Theeuwes, 2010), perhaps by attenuating the response of the transient channel. Another possibility is that the top-down gain for the attended location can be increased so that it exceeds the activity of the transient channel. In this case, intense focus on the current object prevents attentional capture, which is consistent with the psychological concept of the attentional window (Belopolsky and Theeuwes, 2010).

## Discussion

We have proposed a new model of the WTA network that can simultaneously select multiple spatial locations based on a shared feature value. We named the model the feature-based WTA (F-WTA) network because the unit of selection is not a point in space or object, but rather an abstract feature value that is set by the top-down signals. We have demonstrated how the F-WTA network implements the central proposal of the Boolean theory of visual attention that there exists a spatial map that divides the visual space into two mutually exclusive sets. One set represents all locations that are occupied by the chosen feature value. The other set contains all other locations, which are not of interest. The Boolean map controls spatial selection and access to the consciousness (Huang and Pashler, 2007). Moreover, we have shown that the network successfully integrates information across space and time to form the intersection or union of two maps that are defined by different feature cues. Previous models of the WTA network are not capable of such integration because they require that the current winner be externally inhibited to allow attentional focus to move from one location to another (Kaski and Kohonen, 1994; Itti and Koch, 2000, 2001). Another possibility to move activity across locations in the network is to introduce dynamic thresholds that simulate habituation or fatigue in individual neurons. In this case, current winner loses its competitive advantage due to the raise of its threshold. This allows non-winners to gain access to working memory (Horn and Usher, 1990). However, both approaches are not suitable for forming the intersection or union of a set of previous winners and a set of later winners.

Another important property of the F-WTA network that sets it apart from previous models of WTA behavior is the ability to select and store arbitrarily many locations in the memory. This is achieved by inhibitory retrograde signaling, which effectively isolates winning nodes from mutual inhibition. First, the amount of inhibition in the network is significantly reduced because the inhibitory interneuron computes the maximum instead of the sum of the recurrent input that it receives from the excitatory nodes. Second, the winning excitatory nodes release their retrograde signals and block inhibition from the interneuron. Consequently, arbitrarily many winners can participate in representing the selected locations without degrading their activation. In other words, there is no capacity limit on the number of objects that can be simultaneously selected. This is consistent with recent behavioral findings that suggest that our ability to select multiple objects is not fixed. Rather, spatial attention should be considered a fundamentally continuous resource without a strict capacity limit (Davis et al., 2000, 2001; Alvarez and Franconeri, 2007; Liverence and Franconeri, 2015; Scimeca and Franconeri, 2015).

In addition, the network is sensitive to the sudden appearance of a new object in the scene, which suggests that it can also be guided by bottom-up feature cues (Theeuwes, 2013). We hypothesize that the network receives strong input from the transient channel. Such input overrides the network's current memory state, thereby making it sensitive to abrupt onsets. Moreover, the transient channel can be activated by any type of change in the spatiotemporal energy of the input, and not just by the sudden appearance (or disappearance) of objects. For example, it will be activated by a sudden change in the direction of motion (Farid, 2002). When the network simultaneously receives transient input from different locations, they all will be selected together. In this way, the network achieves temporal grouping of synchronous transient input. That is, the network can discover spatial structures that are defined purely by temporal cues (Lee and Blake, 1999; Rideaux et al., 2016).

### Biophysical considerations

As noted above, the model of the F-WTA network rests upon three key computational elements: the dendrite as an independent computational unit, retrograde signaling on synaptic contacts, and computing the maximum over inputs. Here, we review supporting neuroscientific evidence that suggests that all three biophysical mechanisms are plausible candidates for computation in real neural networks.

There is a growing body of evidence that the excitatory pyramidal cell should not be viewed as a single electrical compartment. Rather, it consists of multiple independent synaptic integration zones arranged in a two-layer hierarchy (Häusser and Mel, 2003; London and Häusser, 2005; Branco and Häusser, 2010; Mel, 2016). Using a detailed biophysical model of the pyramidal neuron, Poirazi et al. (2003) showed that its output is well approximated by a two-layer neural network. In the first layer of the network, dendrites independently integrate their synaptic input and produce sigmoidal output. In the second layer, the dendritic output is summed at the soma to produce the neuron's firing rate. Importantly, the somatic and dendritic output functions need not be the same (Jadi et al., 2014). For example, Behabadi and Mel (2014) showed that the soma of the model neuron generates nearly linear output, while the dendritic output is sigmoid. In our model, the dendrite conveys recurrent excitation to the node. Due to the dendritic non-linearity, there is no risk of unbounded activity growth in the node. Furthermore, the dendritic output is summed with the external input at the soma of the node. By using a linear output function at the soma, we have ensured that the F-WTA network remains sensitive to input fluctuations.

Synaptic transmission can be dynamically regulated in an activity-dependent manner, as shown by the existence of depolarization-induced suppression of inhibition (DSI) (Pitler and Alger, 1992) and depolarization-induced suppression of excitation (DSE) (Kreitzer and Regehr, 2001). DSI (DSE) refers to the reduction in inhibitory (excitatory) post-synaptic potentials following depolarization of the postsynaptic cell. These processes have been observed in various brain regions, including the cerebellum, hippocampus, and neocortex. A retrograde messenger that is released from postsynaptic cell due to its depolarization mediates DSI and DSE. After release, the retrograde messenger binds to the receptors at the presynaptic axon terminals and suppresses the release of the transmitter. Based on these properties, Regehr et al. (2009) suggested that a possible physiological function of DSI and DSE is to provide negative feedback that reduces the impact of the synaptic input on the ongoing neural activity.

The model behavior rests upon the assumption that the inhibitory interneuron computes the maximum instead of the sum of its inputs. There is some direct physiological evidence that real cortical neurons indeed compute the MAX function. For example, Sato (1989) examined responses of neurons in the primate inferior temporal cortex to the presentation of one or two bars in their receptive field. He concluded that the responses to two bars that were presented simultaneously were well described by the maximum of the responses to each separately. In a similar vein, Gawne and Martin (2002) recorded the activity of neurons in primate V4 and found that their firing rate in response to the combination of stimuli is best described by the maximum function over the firing rates that are evoked by each stimulus alone. Furthermore, Lampl et al. (2004) directly measured membrane potentials in the complex cells of the cat primary visual cortex and found evidence for the MAX-like behavior in response to the pair of optimal bars.

Indirectly, the importance of the MAX-like operation in cortical information processing can be appreciated by considering the many computational models of visual functions that have employed it in simulating rich and complex datasets. For example, Riesenhuber and Poggio (1999) employed hierarchical computation of the MAX function in a model of invariant object recognition. Spratling (2010, 2011) used it in simulating a large range of classical and non-classical receptive field properties of V1 neurons. Moreover, Tsui et al. (2010) used MAX-like input integration to explain diverse properties of MT neurons and Hamker (2004) used it in his model of top-down guidance of spatial attention. Furthermore, Kouh and Poggio (2008) developed a canonical cortical circuit that is capable of many non-linear operations, including computation of the MAX function. Here, we have shown that a single inhibitory node that is endowed with retrograde signaling can compute the maximum.

Based on the proposed model, we have derived two testable predictions. The cortical network that is involved in spatial selection will contain inhibitory interneurons that can compute the MAX function. Moreover, both the excitatory and inhibitory neurons in this network will be endowed with the anatomical structures that support retrograde signaling (presynaptic receptors and postsynaptic transmitter release sites).

### Comparison with other WTA network models

Several models of biophysical mechanisms have been proposed for implementing WTA behavior in a neural network, including linear-threshold units (Hahnloser, 1998; Rutishauser and Douglas, 2009), non-linear shunting units (Grossberg, 1973; Fukai and Tanaka, 1997), and oscillatory units (Wang, 1999; Borisyuk and Kazanovich, 2004).

A simple model of a competitive network that is based on linear-threshold units has been extensively studied. Stability analysis revealed that this network requires fine-tuning of the connectivity to achieve stable dynamics that can perform cognitively relevant computations, such as choice behavior (Hahnloser, 1998; Hahnloser et al., 2003; Rutishauser et al., 2015). Recently, Binas et al. (2014) showed that a biophysically plausible learning mechanism could tune the network connections in a way that keeps the network dynamics in the stable regime. Here, we have shown how dendritic and synaptic non-linearities ensure that the network dynamics near fixed points depends only on the time constants of the nodes and not on the parameters that control recurrent excitation and lateral inhibition. Therefore, a precise balance between excitation and inhibition is not necessary for achieving a stable memory state. Moreover, the network is sensitive to the input and can iteratively combine the current memory state with new input to form the intersection or union of them.

An important problem for WTA networks that are based on the linear-threshold or sigmoid output functions is that they lack a mechanism for controlling inhibition between the winning nodes. Therefore, they have limited capacity to represent multiple winners. Usher and Cohen (1999) showed that their activation decreases up to the point of complete inactivation as the number of winning nodes increases. This is due to the increased amount of mutual inhibition. The problem cannot be solved simply by reducing the strength of the lateral inhibition because it is not known in advance how many locations will be cued. On the other hand, feature-based spatial selection requires that the network be able to adjust automatically the amount of inhibition to accommodate the selection of a very small or very large number of winners.

Grossberg (1973) proposed a recurrent competitive map model that was based on shunting non-linear interaction between the synaptic input and the membrane potential. The output of the model depends on the exact form of the signal function that is used to convert membrane potential into the firing rate. When the signal function is chosen to grow faster than linear, the network exhibits WTA behavior. By contrast, when the signal function is sigmoid, the network can select multiple winners if they have similar activity levels. The most important property of this model is the existence of the quenching threshold. All nodes whose activity is above QT are enhanced and all nodes whose activity is below QT are suppressed. This behavior is similar to the operation of the F-WTA network that was proposed here. However, an important difference is that in the shunting model, QT is fixed and dependent on the parameters of the network. In contrast, the feature-based WTA network exhibits dynamic QT that depends on the input to the network and not on its parameters. In this way, the F-WTA network rescales its sensitivity to the input fluctuations.

More recently, a version of the recurrent competitive map was applied in modeling object-based attention (Fazl et al., 2009). It was shown that sustained network activity in the model PPC encompasses the whole object as an attentional shroud around it. Such spatial representation of a single object supports view-invariant object recognition within a larger neural architecture, namely, ARTSCAN. In an extension of the model, Foley et al. (2012) proposed two separate competitive networks that account for distinct properties of object- and space-based attention. A network with strong inhibition is limited to the selection of a single object. The other network utilizes weaker inhibition to support multifocal spatial selection. To increase the capacity of this network to represent multiple objects, Foley et al. (2012) suggested that the amount of lateral inhibition could be controlled externally. As the number of objects that should be selected together increases, the lateral inhibition should become weaker to counteract the effect of the larger number of nodes that participate in the competition. In contrast, the F-WTA network does not require such external adjustments of the strength of the lateral inhibition to accommodate the selection of arbitrarily many objects of arbitrary size. Moreover, in the F-WTA network, object-based and multifocal spatial attention coexist within the same circuit. Whether the network exhibits object-based spatial selection depends on the type of cue that is presented to the network and not on its parameters.

Wang (1999) proposed a model of object-based attention that relies on the phase synchronization and desynchronization among oscillatory units. At each location of the recurrent map, there is a pair of excitatory and inhibitory units with distinct temporal dynamics that creates a relaxation oscillator. Excitatory units are also mutually connected with their nearest neighbors and with a global inhibitor. The network is initialized with random phase differences between oscillators at different network locations. The activity of the global inhibitor further enforces phase separation among excitatory units. However, local excitatory interactions among nearest neighbors oppose global inhibition and result in phase synchronization that spreads among nodes that encode the same object. The net result of these interactions is temporal segmentation and selection of one active object representation at a time in a multi-object input image. Importantly, the network can switch its activity from one object representation to another. However, this transition is generated internally by the oscillator dynamics. It is not possible to drive the object selection by external cues such as top-down gain control or bottom-up cues such as abrupt onsets. Moreover, it is not possible to enforce simultaneous selection of more than one object by a joint feature value because the global inhibitor will desynchronize all nodes that encode non-connected items. Therefore, it is not clear how synchronous oscillations could support feature-based attentional selection. Taken together, it is still an open issue whether they are relevant for perception and cognition (Ray and Maunsell, 2015).

### Limitations

The proposed model of spatial selection successfully simulates the formation of the Boolean map and its elaboration by the set operations of intersection and union but does not fully implement all aspects of the theory that was proposed by Huang and Pashler (2007). Precisely, it does not explain why attention is limited to only one feature value per dimension or how the observer sequentially chooses one feature value after another or combines feature dimensions into intersections or unions of Boolean maps. It is likely that this severe limitation arises from some form of the WTA network. However, this constraint requires a more elaborate model of the interactions among the spatially invariant representation of the feature values in the IT cortex and the interactions between the IT and the prefrontal cortex, where decisions and plans are made.

In all simulations that are reported here, we kept items segregated in space. This was not the case in the stimuli that were used by Huang and Pashler (2007). They employed a matrix of colored squares that were connected to one another. This is because activity spreading can occur among adjacent nodes even if they encode different feature values. Activity spreading is observed after top-down signals stop favoring one feature value over the other. In this case, all feature maps contribute equally to the input of the F-WTA network and the network is no longer able to discriminate between selected and unselected feature values. One way to solve this issue is to assume that the top-down signals are constantly present during the whole trial. In this way, the activity magnitude on the cued locations is kept above that on the non-cued locations. Therefore, non-cued locations are treated as background noise and suppressed, despite their proximity to the cued locations. Another possibility is to impose boundary signals that act upon recurrent collaterals of the nodes in the F-WTA network in a way that is similar to how activity spreading is stopped in the network models of brightness perception (Grossberg and Todorović, 1988), visual segmentation (Domijan, 2004), and figure-ground organization (Domijan and Šetić, 2008).

Finally, input to the network does not follow the distance-dependent activity profile that is usually observed in the visual cortex. However, this is not a critical issue for the model's performance because the precision of selection depends on the thresholds for presynaptic terminal activation, namely, *T*_*x*_, and *T*_*y*_. If they are set to very small values, the network will tend to select the centers of the objects when the input pattern is convolved with a Gaussian filter. In contrast, if they are set to larger values, the network will be able to select extended parts of the objects and possibly even the whole objects. In the same way, the model achieves resistance to the input noise. As thresholds are set to larger values, the network can tolerate a larger amount of noise. However, this comes at a cost of less-precise selection, as demonstrated by the simulation that is shown in Figure 12.

## Conclusions

We have demonstrated how the feature-based WTA network achieves spatial selection of all locations that are occupied by the same feature value without suffering from capacity limitations. The network responds to the top-down cue by storing in memory spatial pattern that corresponds to the cued feature value, while non-cued feature values are suppressed. In this way, we have shown how the Boolean map is formed. In addition, we have shown that it is possible to create more complex spatial representations that involve the intersection or the union of two or more Boolean maps. In this way, the F-WTA network goes beyond the capabilities of previous models of the competitive neural network, which cannot integrate information across space and time. Our work suggests that dendritic non-linearity and retrograde signaling are biophysically plausible mechanisms that are essential for model success.

## Author contributions

DD designed the study and write the manuscript. MM performed computer simulations and write the manuscript.

### Conflict of interest statement

The authors declare that the research was conducted in the absence of any commercial or financial relationships that could be construed as a potential conflict of interest. The reviewer, MU, and handling editor declared their shared affiliation.
